# Recruitment of the Complete hTREX Complex Is Required for Kaposi's Sarcoma–Associated Herpesvirus Intronless mRNA Nuclear Export and Virus Replication

**DOI:** 10.1371/journal.ppat.1000194

**Published:** 2008-10-31

**Authors:** James R. Boyne, Kevin J. Colgan, Adrian Whitehouse

**Affiliations:** 1 Institute of Molecular and Cellular Biology, Faculty of Biological Sciences, University of Leeds, Leeds, United Kingdom; 2 Astbury Centre for Structural Molecular Biology, University of Leeds, Leeds, United Kingdom; University of Southern California School of Medicine, United States of America

## Abstract

A cellular pre-mRNA undergoes various post-transcriptional processing events, including capping, splicing and polyadenylation prior to nuclear export. Splicing is particularly important for mRNA nuclear export as two distinct multi-protein complexes, known as human TREX (hTREX) and the exon-junction complex (EJC), are recruited to the mRNA in a splicing-dependent manner. In contrast, a number of Kaposi's sarcoma–associated herpesvirus (KSHV) lytic mRNAs lack introns and are exported by the virus-encoded ORF57 protein. Herein we show that ORF57 binds to intronless viral mRNAs and functions to recruit the complete hTREX complex, but not the EJC, in order assemble an export component viral ribonucleoprotein particle (vRNP). The formation of this vRNP is mediated by a direct interaction between ORF57 and the hTREX export adapter protein, Aly. Aly in turn interacts directly with the DEAD-box protein UAP56, which functions as a bridge to recruit the remaining hTREX proteins to the complex. Moreover, we show that a point mutation in ORF57 which disrupts the ORF57-Aly interaction leads to a failure in the ORF57-mediated recruitment of the entire hTREX complex to the intronless viral mRNA and inhibits the mRNAs subsequent nuclear export and virus replication. Furthermore, we have utilised a trans-dominant Aly mutant to prevent the assembly of the complete ORF57-hTREX complex; this results in a vRNP consisting of viral mRNA bound to ORF57, Aly and the nuclear export factor, TAP. Strikingly, although both the export adapter Aly and the export factor TAP were present on the viral mRNP, a dramatic decrease in intronless viral mRNA export and virus replication was observed in the absence of the remaining hTREX components (UAP56 and hTHO-complex). Together, these data provide the first direct evidence that the complete hTREX complex is essential for the export of KSHV intronless mRNAs and infectious virus production.

## Author Summary

Following gene expression in the nucleus, newly transcribed messenger RNA (mRNA) is exported to the cytoplasm, where it is translated into protein. In mammals the vast majority of mRNAs contain introns that must be removed by the spliceosome prior to nuclear export. In addition to excising introns, splicing is also essential for the recruitment of a several protein complexes to mRNA, one example being the human transcription/export complex, which is required for mRNA export. Herpesviruses, such as Kaposi's sarcoma–associated herpesvirus, replicate by hijacking components of the host cells biological machinery, including those proteins necessary for mRNA export. An intriguing caveat in herpesvirology is that herpesviruses, such as Kaposi's sarcoma–associated herpesvirus, produce some mRNAs that lack introns and do not undergo splicing. How then are these intronless mRNAs exported to the cytoplasm? The answer lies in a virus protein called ORF57 that is able to bind to the intronless mRNA and then export them to the cytoplasm. ORF57 achieves this function by mimicking splicing and recruiting the human transcription/export complex to the intronless viral mRNA, thus facilitating its export into the cytoplasm.

## Introduction

The nuclear export of mRNA composes one part of a larger network of molecular events that begin with transcription of the mRNA in the nucleus and end with its translation and degradation in the cytoplasm. During trafficking to the cytoplasm, a nascent mRNA undergoes numerous co-transcriptional processing steps, including 5′ capping, splicing to remove introns and 3′ polyadenylation [1]–[3]. Of these events it has become clear that splicing is particularly important for mRNA nuclear export [4]. The question of exactly which proteins regulate mRNA nuclear export has been the focus of several recent reviews [5]–[8].

Two distinct multi-protein complexes are recruited to cellular mRNAs as a consequence of splicing, namely the human transcription/export complex (hTREX) and the exon-junction complex (EJC). The hTREX complex contains the proteins Aly (a NXF/TAP-adapter), UAP56 (a RNA-helicase) and the hTHO-complex (a stable complex composed of hHpr1, hTho2, fSAP79, fSAP35 and fSAP24) [9]. A second multi-protein complex, termed the exon-junction complex (EJC) is deposited 20–24 nucleotides upstream of the exon-exon boundary during splicing. Until recently it was believed that Aly and UAP56 were components of the EJC [7], [10]–[12], however, new evidence suggests that Aly and UAP56 are associated exclusively with hTREX and not with the EJC. Therefore, these results suggest that hTREX and EJC are distinct complexes, bind at separate locations on the spliced mRNA [13] and have separate functions, where hTREX directs nuclear export of mRNA and the EJC may instead monitor mRNA fidelity and function during translation [14]–[16].

At present, it is not fully understood what regulates hTREX assembly on the mRNA but in addition to splicing the 5′ cap is also essential for its recruitment [9],[13]. Specifically, an interaction between Aly and the cap-binding complex protein, CBP80 appears to be critical for assembly. Indeed, the 5′ cap has been shown to be required for mRNA export in *Xenopus* oocytes [13]. In contrast to the EJC which binds near each exon-exon boundary, hTREX is recruited exclusively to the 5′ end of the first exon, presumably regulated in part by the reported interaction between CBP80 and Aly [13]. It has been suggested that localising the export proteins at its 5′ end affords the mRNA polarity when exiting the nuclear pore. Therefore, a current model for mRNA export favours a situation where hTREX is recruited to the 5′ cap of spliced mRNA and once bound Aly stimulates the recruitment of the export factor, TAP. TAP then interacts with p15 and the nucleoporins, providing the connection between the ribonucleoprotein (RNP) and the nuclear pore [17]. The functional roles, if any, played by UAP56 and hTHO-complex in this process remain poorly characterised.

Kaposi's sarcoma-associated herpesvirus (KSHV)/Human herpesvirus 8 (HHV8) is a γ-2 herpesvirus associated with a number of AIDS-related malignancies including Kaposi's Sarcoma (KS), primary effusion lymphoma (PEL) and multicentric Castleman's disease [18]–[21]. In contrast to the majority of mammalian genes, a property shared amongst all herpesviruses is that a proportion of lytically expressed viral genes lack introns. Although, KSHV expresses a higher proportion of spliced genes than other herpesviruses, it still encodes a significant proportion of lytically expressed late structural genes which lack introns. KSHV replicates in the nucleus of the host mammalian cell, and therefore requires its intronless mRNAs to be exported out of the nucleus to allow viral mRNA translation in the cytoplasm. This raises an intriguing question concerning the mechanism by which the viral intronless mRNAs are exported out of the nucleus in the absence of splicing. To circumvent this problem, and to facilitate viral mRNA export, herpesviruses of all subfamilies encode a functionally conserved phosphoprotein which has an essential role in viral lytic replication [22]. In KSHV this protein is encoded by the intron-containing open reading frame 57 (ORF57) and has been the subject of several recent reviews [23]–[26]. The ORF57 gene product interacts with Aly, binds viral mRNA, shuttles between the nucleus and the cytoplasm and promotes the nuclear export of viral mRNA transcripts [27]–[31]. These properties are also conserved in ORF57 homologues such as ICP27 from Herpes simplex virus type-1 (HSV-1), SM protein from Epstein Barr virus (EBV) [32]–[35] and the Herpesvirus saimiri (HVS) ORF57 protein [27], [31], [36]–[38].

Here we show that KSHV ORF57 interacts during viral replication with CBP80 and hTREX, but not the EJC. We further show that ORF57 orchestrates the assembly of hTREX onto an intronless viral mRNA. The ORF57-mediated recruitment of hTREX is achieved via a direct interaction between ORF57 and Aly. Furthermore, *in vitro* data showed that UAP56 acts as a bridge between Aly and the hTHO-complex protein hHpr1, thereby facilitating the formation of the complete hTREX complex. When we prevented the recruitment of Aly onto intronless viral mRNA using an ORF57 Aly-binding mutant, this resulted in a failure of ORF57-mediated viral mRNA export and significantly reduced virus replication. Strikingly, expression of a dominant negative Aly mutant that prevented the recruitment of UAP56 and hTHO-complex onto intronless viral mRNA resulted in a dramatic reduction in intronless viral mRNA export and infectious virus production. We therefore propose that the entire hTREX complex must be recruited to intronless viral mRNA by ORF57 in order for efficient intronless mRNA nuclear export and KSHV replication to occur.

## Results

### KSHV ORF57 interacts with CBP80 and the hTREX complex but not the EJC

**Figure 1:**
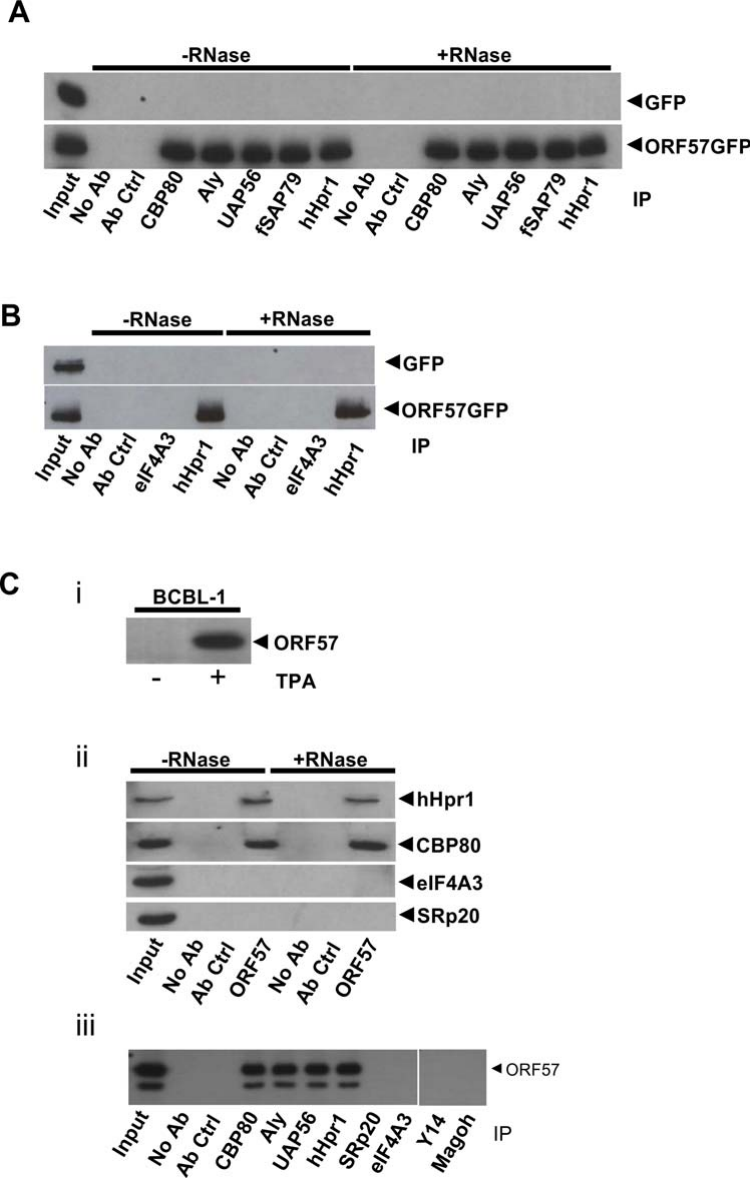
KSHV ORF57 interacts with hTREX. (A) Cells were transfected with pGFP or pORF57GFP and immunoprecipitations performed with the indicated antibody and analysed by western blot using a GFP-specific antibody. Total cell lysate from transfected cells served as positive controls (input). (B) Cells were transfected with pGFP or pORF57GFP and immunoprecipitations performed using hHpr1- and eIF4A3-specific antibodies. Western blot analysis was performed using a GFP-specific antibody. Total cell lysate from transfected cells served as a positive control (input). (C) BCBL-1 cells remained latent or reactivated using TPA, reactivation was confirmed by detection of the ORF57 protein (i). Immunoprecipitations using an ORF57-specific antibody were performed on reactivated BCBL-1 cell lysates that were either untreated or treated with RNase. Western blot analysis was then performed using hHpr1-, CBP80-, eIF4A3 and SRp20-specific antibodies, total reactivated cell lysate served as a positive control (input) (ii). Reactivated BCBL-1 cell lysate was used in immunoprecipitations with CBP80-, Aly-, UAP56-, hHpr1-, SRp20-, eIF4A3-, Y14 and Magoh-specific antibodies. Western blot analysis was performed using an ORF57-specific antibody (iii).

The hTREX complex contains several nuclear export proteins. Given that KSHV ORF57's primary role is attributed to the nuclear export of intronless viral mRNA, we first assessed if ORF57 interacted with hTREX components using co-immunoprecipitation assays. Moreover, as hTREX forms a complex with the 5′-cap protein CBP80 [13], we were interested if ORF57 also interacted with CBP80. 293T cells were transfected with pGFP or pORF57GFP and untreated or RNase treated total cell lysate was used in co-immunoprecipitation experiments with CBP80-, Aly-, UAP56-, fSAP79- and hHpr1- specific antibodies in addition to an unrelated antibody control (a p53-specific antibody). Each of the hTREX proteins and CBP80 co-precipitated with ORF57, in an RNA-independent manner (Fig. 1A). Moreover, indirect immunofluorescence showed that a proportion of ORF57GFP co-localised with hTREX proteins (Fig. S1).

To assess whether ORF57 also interacts with the EJC, co-immunoprecipitation assays were repeated using an antibody specific for eIF4A3, a core EJC component [39] and a hHpr1-specific antibody, serving as a positive control. No interaction was observed with the EJC core component, eIF4A3, in contrast, ORF57 was readily detectable in the hHpr1 immunoprecipitation (Fig. 1B). A control immunoprecipitation was performed to confirm that the eIF4A3 antibody precipitated EJC components (Y14) in this assay (data not shown).

In order to address potential overexpression artefacts and to assess whether ORF57 interacts with hTREX core components during lytic replication, KSHV-latently infected BCBL-1 cells were reactivated using the phorbol-ester, TPA, and lytic gene expression confirmed by detection of the ORF57 protein in TPA-treated cells by western blot analysis (Fig. 1C (i)). Reactivated BCBL-1 cell lysate remained untreated or was treated with RNase and co-immunoprecipitations performed using an ORF57-specific antibody. Western blot analysis using CBP80- and hHpr1- specific antibodies revealed that ORF57 interacts with CBP80 and hHpr1 during lytic replication, however ORF57 did not precipitate with either eIF4A3 (the EJC core component) or the cellular intronless mRNA-export protein, SRp20 (Fig. 1Cii). Moreover, to confirm that ORF57 failed to interact with additional components of the EJC, co-immunoprecipitations were repeated using reactivated BCBL-1 cell lysates and Y14- and Magoh-specific antibodies. Results demonstrate that ORF57 did not precipitate with these additional EJC components (Fig. 1Ciii). A control immunoprecipitation was also performed to confirm that the Y14- and Magoh-specific antibodies precipitated eIF4A3 in this assay (Fig. S2). Therefore, these data provide the first direct evidence of a viral protein associating with CBP80 and all the core components of the hTREX complex.

### ORF57 loads hTREX, but not the EJC, onto intronless viral mRNA transcripts

**Figure 2:**
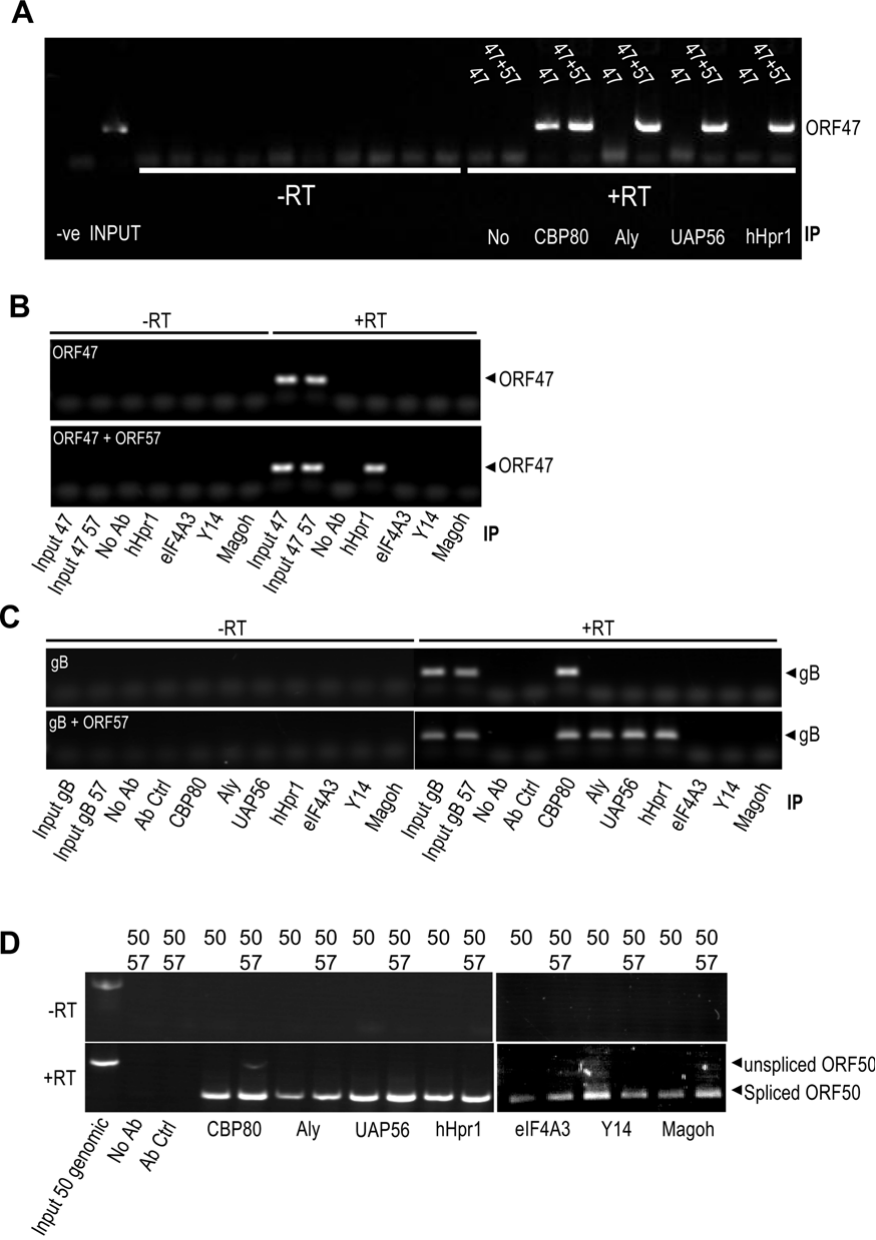
ORF57 recruits hTREX to intronless viral mRNA but does not recruit the EJC. (A) Cells were transfected with pORF47 in the absence or presence of pGFP or pORF57GFP. Following UV crosslinking, RNA-IPs were performed using CBP80-, Aly-, UAP56- and hHpr1-specific antibodies. Total RNA extracted from mock and ORF47 transfected cells served as controls (input). (B) RNA-IPs were repeated using specific antibodies to the EJC components, eIF4A3, Y14 and Magoh, a hHpr1-specific antibody served as a positive control. (C) RNA-IPs were repeated using a second, intronless mRNA reporter, gB. RNA-IPs were performed using the labelled antibodies. Total RNA extracted transfected cells served as controls (input). (D) RNA-IPs were carried out on cells transfected with pORF50 in the absence or presence of ORF57. RNA-IPs were performed using the labelled antibodies. Amplification of the pORF50 vector using ORF50 RT oligos which span the ORF50 intron act as a positive control for PCR.

One possible explanation for how herpesvirus intronless mRNAs undergo nuclear export is that ORF57 mimics splicing by loading key mRNA export proteins, such as hTREX, onto the intronless viral mRNA. In order to test if intronless KSHV transcripts were associated with hTREX proteins and if ORF57 was necessary for this interaction, RNA-immunoprecipitation (RNA-IP) assays were performed. We chose to perform this assay using 2 intronless KSHV mRNAs, specifically ORF47 and gB. RT-PCR and sequence analysis confirmed that both of these ORFs do not contain introns (data not shown). To perform the RNA-IPs, a vector expressing KSHV ORF47 (a late structural intronless gene) was transfected into 293T cells either alone or in the presence of pORF57GFP. Total cell lysates were then used in immunoprecipitations performed with either CBP80-, Aly-, UAP56- or hHpr1-specific antibodies. RNA-IPs performed on cell extracts transfected with ORF47 alone failed to show an interaction between Aly, UAP56 or hHpr1 and the viral ORF47 mRNA (Fig. 2A). In contrast, extracts from cells transfected with both pORF47 and pORF57GFP displayed a clear interaction between Aly, UAP56 and hHpr1 and the intronless viral ORF47 mRNA (Fig. 2A). CBP80 was found to bind to the intronless ORF47 viral mRNA independently of ORF57 (Fig. 2A). Moreover, this analysis was repeated with a second intronless KSHV mRNA, namely the late structural glycoprotein gB, and similar results were observed (Fig. 2C). These data show that ORF57 is required for the recruitment of core components of hTREX onto intronless viral mRNA.

To determine whether EJC components are recruited to intronless viral transcripts prior to export, RNA-IP assays were also performed using eIF4A3-, Y14- and Magoh-specific antibodies. Results failed to show any interaction between the EJC core components and viral intronless ORF47 and gB mRNAs in the absence or presence of ORF57 (Fig. 2B and 2C). These results show that the EJC is not recruited to intronless viral transcripts by ORF57 and suggests that the EJC is not required for KSHV intronless viral mRNA nuclear export.

To determine whether the hTREX and EJC components were recruited to a spliced viral transcript, RNA-IPs were also performed using a vector expressing the genomic (intron-containing) KSHV ORF50 gene. 293T cells were transfected with pORF50 in the absence or presence of ORF57. Total cell lysates were then used in immunoprecipitations performed with either CBP80-, Aly-, UAP56-, hHpr1-, eIF4A3-, Y14- or Magoh-specific antibodies. Results demonstrated that CBP80, hTREX and EJC components were recruited to the spliced ORF50 mRNA in an ORF57 independent manner (Fig 2D). This suggests that splicing of a viral transcript is sufficient to recruit the cellular proteins necessary for nuclear export. In contrast, ORF57 is required for the recruitment of the hTREX proteins to an intronless viral transcript.

### UAP56 functions as a bridge between Aly and the hTHO-complex to facilitate assembly of hTREX

**Figure 3:**
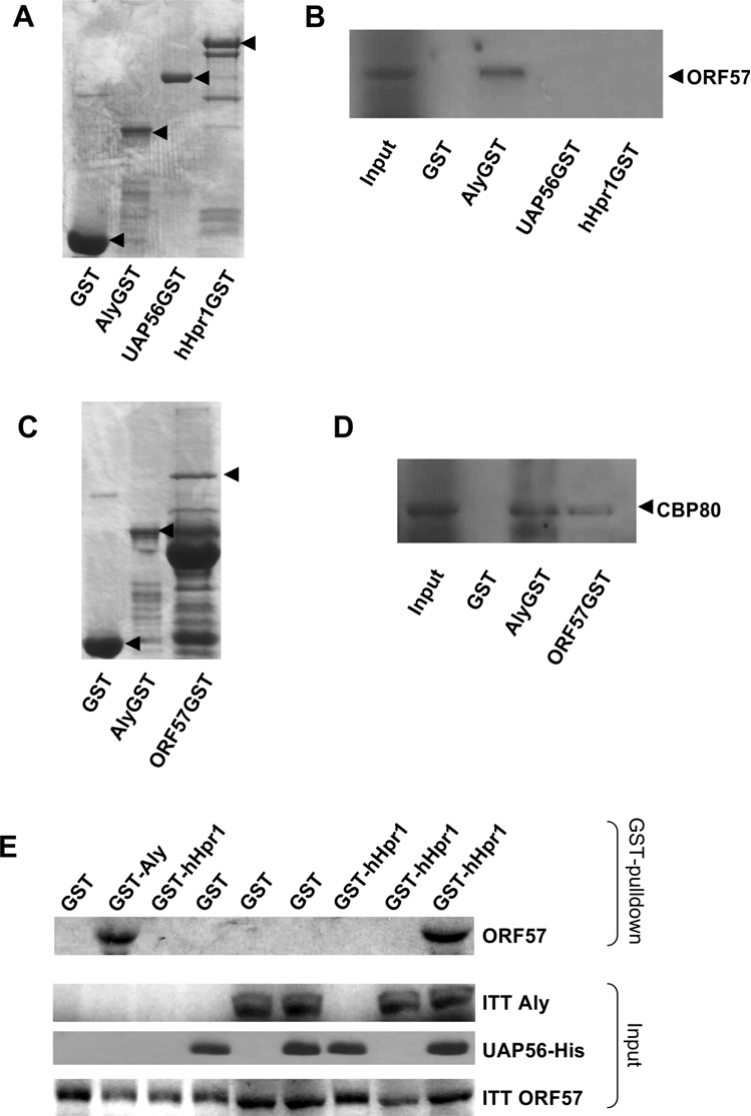
ORF57-hTREX complex formation requires both Aly and UAP56. (A) Equal amounts of recombinant GST, GST-Aly, GST-UAP56, GST-hHpr1 bound to beads were separated by SDS-PAGE and proteins visualised by coomassie staining. (B) Bound recombinant GST-fusion proteins were incubated with ^35^S-Met-labeled ORF57 produced by ITT. Following washes, bound proteins were separated by SDS-PAGE and the dried gel was exposed to autoradiograph film for 16 hrs. (C) Recombinant GST, GST-Aly, GST-ORF57 were bound to glutathione-agarose beads. Following washes, 10% of the beads used in the subsequent GST pull-down assays were separated by SDS-PAGE and proteins visualised by coomassie staining. (D) Bound recombinant GST-fusion proteins were incubated with ^35^S-Met-labeled CBP80 produced by ITT. Following washes, bound proteins were separated by SDS-PAGE and the dried gel was exposed to autoradiograph film for 16 hrs. (E) Bead bound recombinant GST, GST-Aly and GST-hHpr1 fusion proteins were incubated with ^35^S-Met-labeled ORF57 produced by ITT in the absence or presence of ^35^S-Met-labelled Aly or purified His-tagged UAP56. Following washes, bound proteins were separated by SDS-PAGE and the dried gel was exposed to autoradiograph film for 16 hrs. Inputs for ORF57, Aly and UAP56 are shown.

Currently, while it is known that hTREX recruitment to a mammalian mRNA is both 5′-cap- and splicing-dependent, the protein-protein interactions that govern assembly of the hTREX complex itself are not fully understood. As ORF57 functions to recruit hTREX onto the intronless viral mRNA in a splicing independent manner we assessed whether this viral-system could be used to investigate hTREX assembly in more detail. To this end, we sought to determine if any hTREX proteins directly interacted with ORF57. Radio-labelled ORF57 was generated by *in vitro* coupled transcription/translation (ITT), RNase treated, and used in GST pull-down experiments using constructs expressing GST-, GST-Aly, GST-UAP56 and GST-hHpr1 fusion proteins. Equal amounts of each expressed protein were used in each pulldown experiment (Fig. 3A). Analysis showed that ORF57 bound directly to GST-Aly but not to any other hTREX component (Fig. 3B). Due to the instability of GST-CBP80, a reverse pulldown experiment was performed using GST-ORF57 (Fig. 3C) and radio-labelled ITT CBP80, a GST-Aly pulldown with ITT CBP80 served as a positive control [13]. Results also revealed a direct interaction between CBP80 and KSHV ORF57 (Fig. 3D).

These data suggest that ORF57 only interacts directly with Aly and CBP80, therefore the question remains how the complete hTREX complex associates with ORF57. It has previously been suggested that the hTREX complex is formed by UAP56 bridging the interaction between Aly and the hTHO-complex [9]. Therefore, to further investigate ORF57-hTREX assembly, we assessed which hTREX components were required to reconstitute the ORF57-hHpr1 interaction. GST pulldown experiments were performed using GST-hHpr1 and ITT ORF57 alone or combinations with ITT Aly or recombinant UAP56. When the GST-hHpr1 ITT ORF57 pulldown was repeated in the presence of both ITT Aly and purified UAP56, analysis revealed a clear interaction between hHpr1 and ORF57 (Fig. 3E), suggesting that ORF57 requires both Aly and UAP56 to recruit the hTHO-complex, thus facilitating formation of the ORF57-hTREX complex. These findings provide the first direct evidence that UAP56 functions as a bridge between Aly and the hTHO-complex component hHpr1 to facilitate assembly of hTREX. However, at present we cannot exclude the possibility that ORF57 interacts directly with other hTHO-complex components.

### hTREX recruitment to intronless viral mRNA is essential for their nuclear export

**Figure 4:**
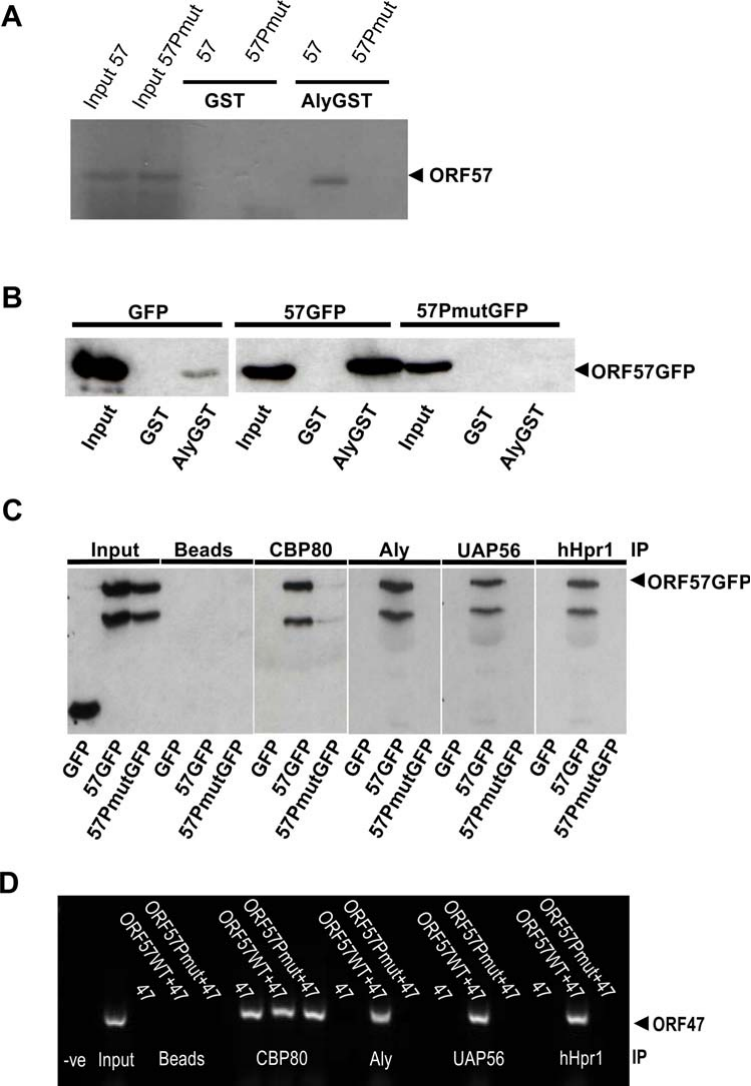
The ORF57 PxxP motif is required for direct interaction with Aly. (A) Recombinant GST and GST-Aly were bound to glutathione-agarose beads and incubated with ^35^S-Met-labeled ORF57 or ORF57Pmut produced by ITT. Following washes, bound proteins were separated by SDS-PAGE and the dried gel was exposed to autoradiograph film for 16 hrs. ITT input controls for ORF57 and ORF57Pmut are shown. (B) Recombinant GST- or GST-Aly-bound glutathione-agarose beads were incubated with pGFP-, pORF57GFP or pORF57PmutGFP-transfected cell extracts. Following washes, bound proteins were analysed by western blot using a GFP-specific antibody. Total cell lysates from transfected cells served as a positive control (input). (C) Cells were transfected with pGFP, pORF57GFP or pORF57PmutGFP and immunoprecipitations performed with the indicated antibody and analysed by western blot using GFP-specific antibody. Total cell lysate from transfected cells served as positive controls (input). (D) RNA-IPs were performed using the indicated antibodies and nested RT-PCR carried out on the extracted RNA using ORF47-specific oligonucleotides. Total RNA extracted from transfected cells served as control (input).

To assess whether hTREX is essential for viral mRNA nuclear export we produced an ORF57 mutant protein which was unable to interact with Aly and as such would be predicted to prevent the recruitment of the complete hTREX complex onto intronless viral mRNA. A minimal region responsible for Aly-binding has been identified in ORF57 and spans 35aa between residues 181 and 215 [28]. Upon closer examination of this sequence, we identified a PxxP-polyproline motif. To assess whether this motif was important for Aly-binding, both proline residues were substituted with alanine residues by site-directed mutagenesis to generate pORF57PmutGFP. To determine if mutating the PxxP-motif in ORF57 led to a loss of Aly binding, GST-Aly pulldown assays were performed using ITT ORF57 or ITT ORF57Pmut. Results demonstrated that the mutant ORF57 protein was unable to interact with GST-Aly, in contrast to the wild type protein (Fig. 4A). Moreover, similar results were observed using pull-down assays with pGFP-, pORF57GFP- or pORF57PmutGFP-transfected 293T cell lysates (Fig. 4B). These data demonstrate that the ORF57 PxxP-motif is required for the direct interaction with Aly. To confirm that the mutagenesis of the PxxP motif had no effect on ORF57 protein stability or other reported functions, several independent experiments were performed to assess the ability of ORF57PmutGFP to localise to nuclear speckles, homodimerise, directly interact with ORF50 and bind viral intronless mRNA (Fig. S3), all of which are features of the wild type ORF57 protein. In each case the ORF57PmutGFP phenotype was indistinguishable from that of wild type ORF57.

Having established that ORF57PmutGFP is unable to interact with Aly and that the mutation does not affect other ORF57 functions, we then asked if, in the absence of Aly-binding, ORF57 was still able to complex with CBP80 and hTREX components. 293T cells were transfected with pGFP, pORF57GFP or pORF57PmutGFP and total cell lysates were used in co-immunoprecipitation experiments, using CBP80-, Aly-, UAP56-, and hHpr1-specific antibodies. In each case the hTREX antibody immunoprecipitated ORF57GFP but not ORF57PmutGFP, demonstrating that in the absence of the Aly-interaction ORF57 was unable to form a complex with hTREX (Fig. 4C). In addition, the ORF57PmutGFP exhibited a reduced but specific binding to CBP80 (Fig. 4C). This reduced binding may be due to the mutation of the PxxP-polyproline motif either affecting CBP80 binding directly or the loss of hTREX binding affects the stability of the CBP80-ORF57 complex. To further investigate whether the mutation of the PxxP-polyproline motif affected direct binding to CBP80, GST pulldown assays were performed using GST-ORF57 and GST-ORF57PmutGFP. Equal amounts of each expressed protein was incubated with radio-labelled ITT CBP80. Results demonstrated that ORF57 and ORF57PmutGFP bound to CBP80 with similar affinity (Fig. S4). This suggests that the reduced binding observed between ORF57PmutGFP and CBP80 may be due to the loss of hTREX, which is possibly required to stabilise the export competent vRNP.

To determine if ORF57PmutGFP was unable to recruit hTREX proteins to KSHV intronless mRNA transcripts in the absence of Aly binding, RNA-IP assays were performed using CBP80-, Aly-, UAP56- or hHpr1-specific antibodies. These data demonstrate that in contrast to pORF57GFP, pORF57PmutGFP is unable to recruit hTREX components to intronless viral mRNA (Fig. 4D). This suggests that a direct interaction between Aly and ORF57 is required for hTREX recruitment onto intronless viral transcripts.

**Figure 5:**
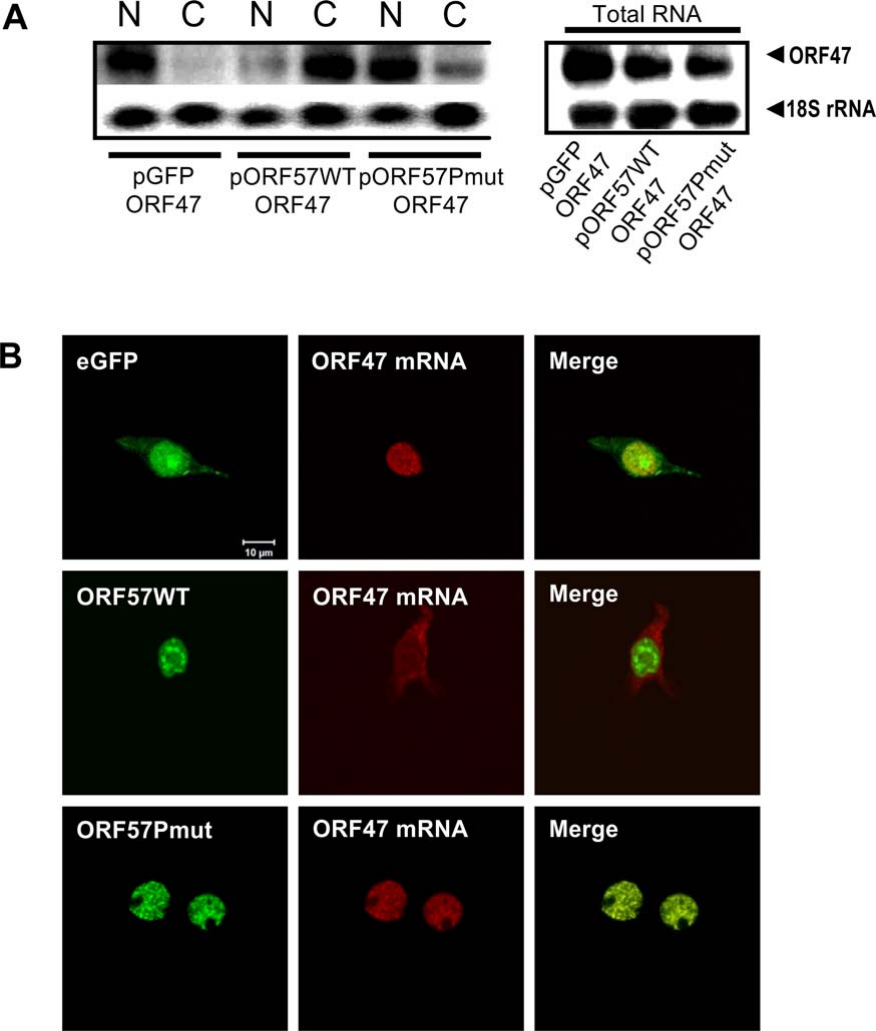
hTREX recruitment to intronless viral mRNA is required for efficient nuclear export. (A) Cells were transfected with pORF47 in the presence of pGFP, pORF57GFP or pORF57PmutGFP and incubated for 24 h. Total RNA or RNA isolated from nuclear and cytoplasmic fractions was analysed by northern blot using an ORF47-specific radio-labelled probe. A probe to the 18S subunit of ribosomal RNA was used as a loading control. Densitometry analysis of three independent northern blot experiments determined relative levels of nuclear, cytoplasmic and total RNAs and standard error calculated (n = 3). (B) 293T cells were transfected with pORF47 in the presence of pGFP, pORF57GFP or pORF57PmutGFP. Cells were then fixed in paraformaldehyde and extracted in SDS buffer. *In situ* hybridisation was subsequently performed using a biotinylated oligonucleotide probe specific to ORF47 mRNA and the probe detected using Cy5-streptavidin. Images shown are a representative of all transfected cells.

To test if a failure in ORF57-mediated recruitment of hTREX to the intronless ORF47 mRNA prevented nuclear export of intronless KSHV transcripts, two independent mRNA export assays were performed. Firstly, northern blotting was used to detect if intronless ORF47 mRNA was present in the nuclear or cytoplasmic fraction of transfected cells. Very little ORF47 mRNA was detected in the cytoplasmic RNA fraction of cells transfected with pORF47 alone (9.9±4.9%), whereas cells co-transfected with pORF47 and pORF57GFP displayed a clear shift in ORF47 mRNA from the nuclear to the cytoplasmic fraction (81.5±1.0%), indicative of ORF57-mediated viral mRNA nuclear export. However, upon co-transfection with pORF47 and pORF57PmutGFP, the majority of ORF47 mRNA was no longer found in the cytoplasmic fraction (21.3±3.8%), instead it was retained in the nuclear pool at similar levels to those seen for the negative control, symptomatic of a failure in ORF57-mediated viral mRNA nuclear export (Fig. 5A). To confirm that the ORF57 mutant did not affect mRNA stability, total RNA levels were assessed by northern blot analysis. No significant difference in ORF47 mRNA levels was observed between cells expressing wild type or mutant ORF57 proteins (Fig. 5A, right panel). However, a slight decrease in total mRNA levels is seen in the presence of both the ORF57 or ORF57PmutGFP compared to the GFP control. At present, the reason for this is unknown, however, it could be due to the overexpression of the ORF57 protein.

To confirm the above result, a fluorescent *in situ* hybridisation assay was utilised. 293T cells were transfected with pORF47, in addition to either pGFP, pORF57GFP or pORF57PmutGFP. 24 h post-transfection cells were fixed, permeabilised and incubated with a biotin-labelled oligonucleotide specific for the KSHV ORF47 mRNA. After a 4 hr hybridisation cells were washed and ORF47 mRNA subcellular localisation was visualised using Cy5-streptavidin. Cells transfected with pORF47 and GFP retained the ORF47 mRNA in the nucleus, whereas ORF47 mRNA was clearly visualised in the cytoplasm of cells transfected with pORF47 and pORF57GFP. However, upon transfection with pORF57PmutGFP, ORF47 mRNA was only observed in the nucleus, symptomatic of a failure in ORF57-mediated viral mRNA nuclear export (Fig. 5B). Together, these two independent assays demonstrate that the ORF57-dependent recruitment of hTREX to intronless viral transcripts is essential for their efficient nuclear export.

We were also interested to determine whether the recruitment of the complete hTREX complex is required for virus replication and infectious virion production. To this end, we utilised a 293T cell line harbouring a recombinant KSHV BAC36-GFP genome [40]. This KSHV-latently infected cell line can be reactivated releasing infectious virus particles in the supernatant which can subsequently be harvested and used to infect 293T cells [41]. The 293T-BAC36 cell line was transfected with pGFP, pORF57GFP or pORF57PmutGFP and concurrently reactivated using TPA and incubated for 72 hours. The supernatants from each flask were then harvested and used to re-infect 293T cells and GFP positive cells were scored 48 h post-infection, as described above. Results revealed similar levels of lytic replication and virus production from cells expressing pGFP or pORF57GFP. However, virus production was significant reduced (P = 0.018) upon the expression of the ORF57PmutGFP (Fig. S5). Therefore, these results demonstrate that the ORF57-dependent recruitment of the complete hTREX complex to intronless viral transcripts is essential for efficient virus lytic replication and infectious virion production.

### ORF57-mediated recruitment of Aly and TAP to intronless viral mRNA is not sufficient for efficient nuclear export and virus replication

The above data show that ORF57 binds viral intronless mRNA and directly interacts with Aly. Given that Aly is able to recruit the export factor TAP directly, it was of interest to determine if UAP56 and the hTHO-complex are required for viral mRNA export. In contrast to the cellular mRNA model, a major advantage of our viral system is that hTREX assembly on the viral mRNA is dependent upon an interaction with a virus-encoded protein, not splicing. Specifically, ORF57 binds viral mRNA, directly interacts with and recruits Aly which in turn then interacts with and uses UAP56 to bridge an interaction with the hTHO-complex. This ordered recruitment allows us to specifically disrupt the viral mRNA-ORF57-hTREX complex at different points and assess the functional significance on nuclear export. Furthermore, rather than using an artificial *in vitro* assay to investigate the functional significance of hTREX, we assessed this in the context of the virus replication cycle using the 293T-BAC36 assay described above.

**Figure 6:**
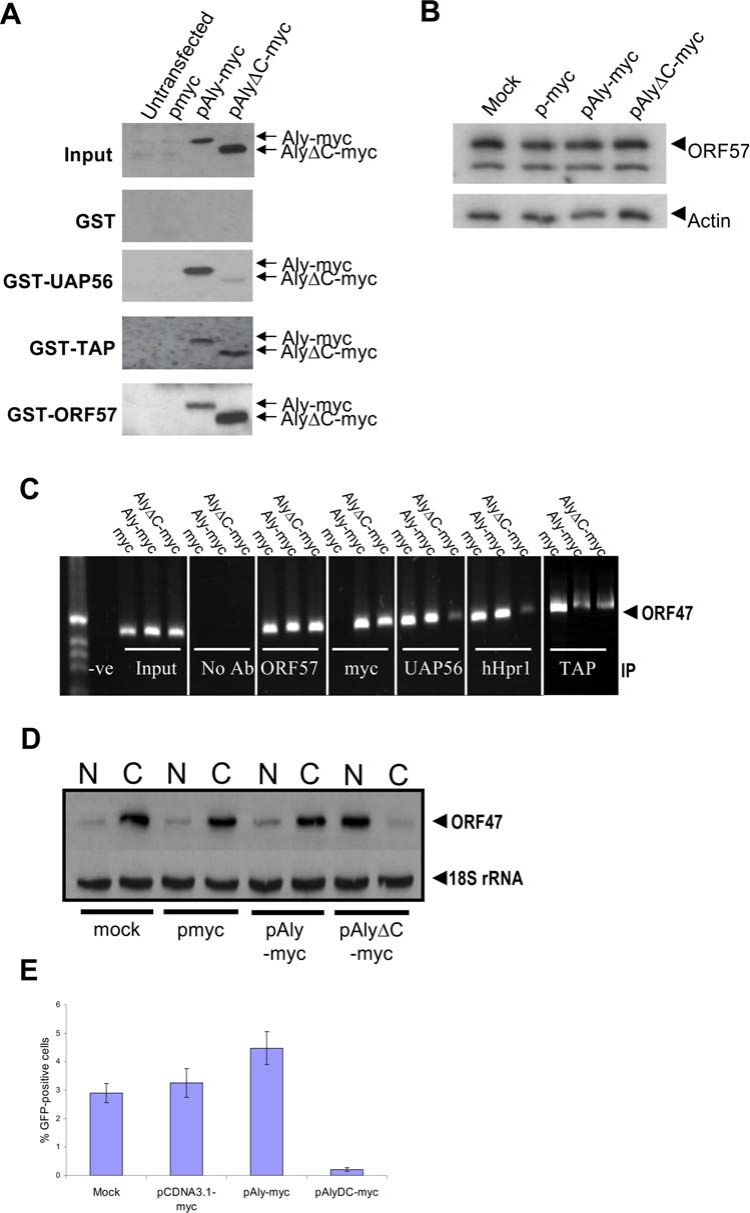
ORF57-mediated recruitment of Aly and TAP to intronless viral mRNA is not sufficient for efficient nuclear export and virus replication. (A) Recombinant GST-, GST-UAP56, GST-TAP or GST-ORF57-bound glutathione-agarose beads were incubated with mock-, pmyc-, pAly-myc- or pAlyΔC-myc-transfected cell extracts. Following washes, bound proteins were analysed by western blot using a myc-specific antibody. Total cell lysate from mock and transfected cells served as a positive control (input). (B) Total cell lysate from mock-, pmyc-, pAly-myc- or pAlyΔC-myc-transfected and reactivated 293T BAC36 cells were isolated at the 24 h time-point and analysed by western blot using an ORF57-specific antibody. Western blot analysis of B-actin levels served as a loading control. (C) RNA-IPs were performed using the indicated antibodies. (D) 293T BAC36 cells were transfected with the indicated vectors and concurrently reactivated using TPA. Northern blot analysis was performed using an ORF47-specific radio-labelled probe. (E) Lytic virus replication was assayed by harvesting the supernatant of transfected 293T BAC36 cells. Supernatant was used to infect 293T cells and 48 h later the level of virus infection was scored by direct-immunofluorescence, n = 3000.

The trans-dominant mutant, pAlyΔC-myc, which has 20 residues deleted from the carboxy-terminus of Aly, is unable to interact with UAP56 [42]. We were interested in establishing if this mutant could be used to disrupt the assembly of UAP56 and hTHO-complex on an intronless viral mRNA and as such provide insights into whether these proteins are essential for nuclear export. However, prior to its use in the replication assay it was essential to confirm that AlyΔC-myc is still recruited by ORF57 to intronless viral mRNA and is able to interact with TAP. To this end, ORF57, UAP56 and TAP were expressed as GST fusion proteins and incubated with either pmyc, pAly-myc or pAlyΔC-myc transfected cell lysates and pulldown analysis performed. Western blotting using a myc-specific antibody demonstrated that Aly-myc interacted with ORF57, TAP and UAP56. In contrast, AlyΔC-myc is unable to associate with UAP56 but retains the ability to interact with both ORF57 and TAP (Fig. 6A). These results suggest that AlyΔC-myc is an ideal mutant to inhibit the recruitment of UAP56 and hTHO-complex on the viral intronless mRNA. However, one caveat to this system is that expression of pAlyΔC-myc may also act in a dominant negative capacity to inhibit spliced mRNA nuclear export [42]. Therefore it was important to allow expression of the spliced ORF57 protein prior to accumulation of pAlyΔC-myc. To this end, transient transfection of pAlyΔC-myc was performed concurrent with reactivation of the KSHV lytic replication cycle, and ORF57 protein levels assessed 24 h later. Results show that comparable amounts of ORF57 were expressed in untransfected, pmyc, pAly-myc and pAlyΔC-myc transfected cell lysates (Fig. 6B).

To test if AlyΔC-myc inhibited the recruitment of UAP56 and the hTHO-complex onto KSHV intronless RNAs, RNA-IPs were performed on reactivated KSHV-infected 293T cells transfected with pmyc, pAly-myc or pAlyΔC-myc. We obtained similar results for pmyc and pAly-myc to those shown in Fig. 2A, where recruitment of hTREX components onto the viral RNA was readily detected 48 h-post reactivation. However, RNA-IPs using cell extracts transfected with AlyΔC-myc showed a dramatic decrease in the recruitment of UAP56 and hHpr1 to viral mRNA (Fig. 6C). RNA-IPs performed using a TAP-specific antibody showed that TAP is recruited to the intronless viral mRNA, irrespective of Aly status. Critically, RNA-IPs using an ORF57-specific antibody produced ORF47 RT-PCR products of a similar intensity, suggesting that ORF57 was not limiting in this assay (Fig. 6C). It should also be noted that we observed a decrease in TAP recruitment to the viral mRNA in the presence of both pAly-myc and pAlyΔC-myc, compared to pmyc control. At present, we are unsure why TAP recruitment is reduced, however, no difference in mRNA nuclear export is observed between pmyc and pAly-myc transfected cells, suggesting that this reduction in TAP recruitment does not impede the nuclear export of intronless viral mRNAs.

To assess if the AlyΔC-myc mutant affected intronless viral mRNA export during replication, northern blot analysis was performed as described above. Results demonstrated that ORF47 mRNA nuclear export is impaired in reactivated cells that expressed AlyΔC-myc, but not in cells expressing myc or Aly-myc (Fig. 6D). Moreover, to determine if expression of AlyΔC-myc had any effect on virus replication, the KSHV-latently infected 293T BAC36-GFP cell line was transfected with pmyc, pAly-myc or pAlyΔC-myc and concurrently reactivated using TPA and incubated for 72 hours. The supernatants from each flask were then harvested and used to re-infect 293T cells. The level of virus replication was determined by scoring the percentage of GFP positive cells 48 h post-infection, as previously described [41]. Similar levels of lytic replication and virus production were observed from pmyc and pAly-myc pre-transfected cells. Strikingly, virus production from pAlyΔC-myc pre-transfected cells was reduced by approximately 10 fold (Fig. 6E). These data demonstrate that ORF57-mediated recruitment of Aly and TAP to an intronless viral mRNA is insufficient for its nuclear export and that a lack of UAP56 and hTHO-complex on an intronless viral mRNA has a profound effect on intronless nuclear export and KSHV lytic replication.

## Discussion

Two distinct multi-protein complexes have been reported to contain export adapter proteins and both are recruited to pre-mRNAs during splicing, namely hTREX and the EJC [6],[8]. A recent report showed that hTREX is recruited exclusively to the 5′ end of the first exon in a splicing- and 5′ cap-dependent manner [13]. In contrast to higher eukaryotes, analysis of herpesvirus genomes has highlighted that a proportion of lytically expressed viral genes lack introns. Herpesviruses replicate in the host cell nucleus and therefore require their intronless mRNAs to be exported out of the nucleus via cellular export pathways. How exactly herpesviruses assemble an export competent intronless mRNA is poorly understood. Here we show that a KSHV encoded protein, ORF57, specifically binds, and subsequently recruits, the hTREX complex, but not the EJC, to intronless viral mRNA. Specific disruption of the ORF57 interaction with hTREX abolishes efficient viral mRNA export. Furthermore, uncoupling of hTREX assembly, demonstrates that recruitment of Aly and TAP alone is not sufficient for intronless viral mRNA nuclear export and virus replication.

### hTREX but not the EJC is recruited to intronless viral mRNA

Co-immunoprecipitation data show that ORF57 readily associates with components of hTREX, however, no such interaction was observed between ORF57 and the EJC proteins; eIF4A3, Y14 and Magoh. This result suggests that the EJC is not recruited to intronless viral transcripts and this was confirmed using RNA-IP assays. In contrast, hTREX proteins readily precipitated with intronless viral mRNA, in the presence of ORF57, which presumably functions as a linker between hTREX and the viral mRNA. These findings suggest that the essential export adapter complex for intronless KSHV nuclear export is hTREX and not the EJC. It should be noted that these findings are in contrast to previous observations using a homologue of KSHV ORF57 from the prototype γ-2 herpesvirus, Herpesvirus saimiri [29]. One possible explanation for these contrasting data is that co-immunoprecipitations from Williams *et al.* were performed by over-expressing myc-tagged EJC components, whereas this in study, endogenous EJC proteins was precipitated using an eIF4A3-, Y14 and Magoh-specific antibodies. To test this, we have performed co-immunopreciptations with EJC specific-antibodies using HVS-infected cell lysates. No interactions were observed between HVS ORF57 and the endogenous EJC proteins (Fig. S6), suggesting the previously observed interactions may have been due to the overexpression of the EJC components. In addition to splicing dependency, the cap-binding complex protein, CBP80, is required to recruit hTREX to human pre-mRNA, via a direct interaction with Aly. Interestingly, we detected a direct interaction between ORF57 and CBP80, implying that the 5′ cap may also function in intronless KSHV mRNA export. However, upon disrupting the ORF57 and Aly interaction (via mutation of the PxxP motif), we also observed a reduction of the ORF57-CBP80 interaction. Analysis suggests that this reduction maybe due to the loss of hTREX affecting the stability of the export competent viral RNP. This suggests that although ORF57 interacts directly with Aly and CBP80, these interactions may not overlap and more detailed analysis of the interacting domains for both proteins is required. It is also worth noting however, that in the absence of ORF57, CBP80 did not recruit Aly to the intronless viral transcripts, suggesting that ORF57 is essential for the loading of hTREX on viral mRNA. The lack of EJC recruitment to intronless viral mRNA may have ramifications beyond those of nuclear export, for example, the EJC has been suggested to function in translational efficiency [16],[43]. Intriguingly, the herpes simplex virus type-1 (HSV-1) ORF57 homologue, ICP27, has been implicated in increased translation efficiency [44],[45], we are currently investigating whether ORF57 increases translation of KSHV transcripts during virus replication.

### Assembly of the complete hTREX complex on viral intronless mRNA is essential for nuclear export and virus replication

**Figure 7:**
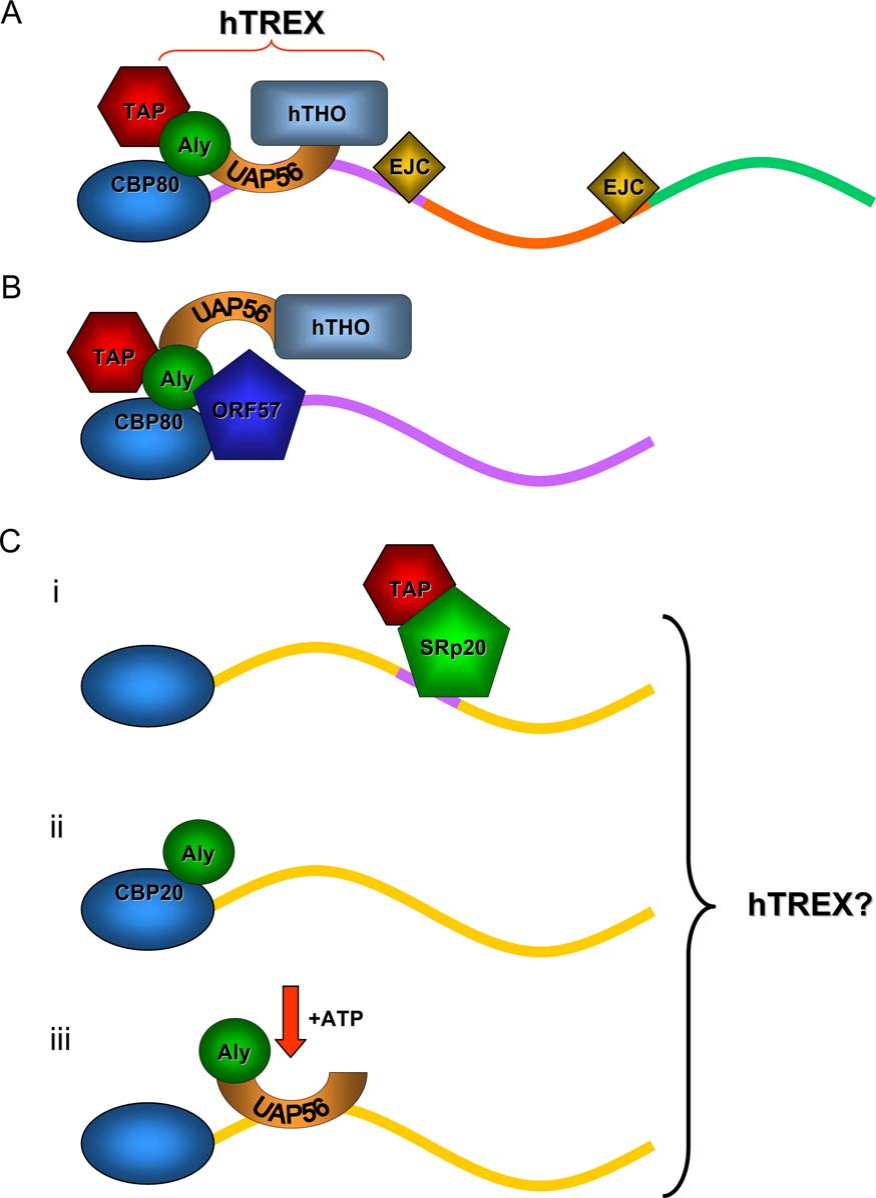
Models for hTREX assembly. (A) hTREX is recruited to spliced mRNA in a 5′ cap- and splicing dependent manner. (B) Our model for hTREX assembly on an intronless viral mRNA. The hTREX complex is recruited via a direct interaction between ORF57 and Aly. (C) Several models for intronless mammalian mRNA export. (i) Huang *et al* report that certain intronless mRNAs are targeted by SR-proteins that recognise specific sequences in the mRNA then directly access the TAP/p15 export factor. (ii) The Nojima *et al* model suggests that Aly is recruited via an interaction with the CBC. (iii) Taniguchi *et al* report that Aly is loaded onto the intronless mRNA by UAP56 in an ATP-driven mechanism.

The current model for hTREX assembly on a spliced mRNA describes UAP56 and Aly associating with the mRNA in a 5′ cap- and splicing-dependent manner. Moreover, as shown in Fig. 7A, it has been suggested that UAP56 may bridge an interaction between Aly and the hTHO-complex [9],[42]. In contrast, during KSHV replication hTREX appears to be tethered to an intronless KSHV mRNA via an exclusive interaction with ORF57. Taking advantage of this, we used the ORF57-hTREX complex to gain insight into how individual components of hTREX interact with one another. Our data show that ORF57 interacts exclusively with Aly, which then binds directly to UAP56 and this in turn functions as a bridge to recruit hHpr1 and presumably the complete hTHO-complex (Fig. 7B). This order of hTREX assembly is in broad agreement with the model proposed by Cheng *et al* who showed using RNase H digestion analysis that Aly was the most 5′ of the hTREX components, with UAP56 and hTHO-complex binding further downstream. Interestingly, the direct interaction observed between ORF57 and CBP80 suggest that ORF57 may recruit hTREX to the 5′ end of the intronless mRNA, perhaps to provide directionality to nuclear export as is the proposed case for spliced human mRNA [13].

The functional significance of hTREX recruitment to intronless viral mRNA is substantiated using an ORF57 point mutant and a dominant-negative Aly mutant. Specifically, we were able to disrupt the direct interaction between ORF57 and Aly by mutating two proline residues within a region of ORF57's Aly-binding domain [28]. This ORF57Pmut was still able to recognise and bind intronless viral mRNA, however, it lacked the ability to recruit hTREX to these transcripts. A failure to recruit hTREX rendered the ORF57Pmut non-functional as a viral mRNA export protein and provides direct evidence that the hTREX complex is essential for the efficient export of intronless viral mRNA and virus replication. The export adapter Aly is able to interact directly with the export factor complex TAP/p15 [46], therefore, we were interested in assessing whether Aly-TAP/p15 recruitment produced an export-competent intronless viral mRNP or if UAP56 and the hTHO-complex were also required for nuclear export. This is of particular importance as a number of ORF57 homologues, such as Herpes simplex virus-type 1 ICP27, have been shown to interact with Aly and TAP, but it is unknown whether they also recruit UAP56 and the hTHO-complex [32],[33]. One major advantage of using the KSHV system to study hTREX assembly in contrast to analysing hTREX recruitment to a spliced human mRNA is that recruitment of hTREX on an intronless viral transcript is mediated via a direct interaction between ORF57 and Aly, which serves to target the remainder of hTREX to the intronless viral mRNA. This facilitated the use of a trans-dominant Aly mutant, termed AlyΔC, which retains a direct interaction with ORF57 yet fails to interact with UAP56. The AlyΔC mutant has limited use as a tool for dissecting hTREX recruitment to spliced human mRNA as it does not bind to spliced mRNA [42]. The introduction of the dominant-negative AlyΔC mutant into a KSHV virus replication system dramatically reduced the amount of UAP56 and hHpr1 recruited to intronless viral transcripts and this in turn led to a striking reduction in intronless viral mRNA nuclear export and significantly, virus replication. Importantly, RNA-IP analysis of intronless viral mRNPs from cells expressing AlyΔC revealed that ORF57, Aly and TAP where all present on intronless viral mRNA, suggesting that UAP56 and perhaps the hTHO-complex possess an unidentified, yet essential role in mRNA nuclear export.

These data place hTREX at the hub of human mRNA nuclear export. However, RNAi studies in *Drosophila melanogaster* and *Caenorhabditis elegans* have shown Aly to be non-essential for mRNA export in these systems [47],[48]. In addition, a genome-wide RNAi study in D. melanogaster reported that the conserved THO-complex was only required by a subset of transcripts for nuclear export [49]. Interestingly, D. melanogaster and C. elegans do require UAP56 both for viability and for bulk mRNA nuclear export [10],[50]. This suggests that while there may be redundancy in eukaryotic systems for certain TREX components, others remain essential. Similar controversy surrounds the role of Aly in herpesvirus mRNA export. In contrast to our data, which show the ORF57-Aly interaction to be essential for efficient intronless viral mRNA nuclear export, a study reported that depletion of Aly using RNAi had little effect on ORF57-mediated transactivation [51]. In addition, a second manuscript reports that differences in the ORF57-Aly binding affinity does not effect ORF57 export function [52]. One possible explanation for these discrepancies is that only partial depletion of Aly was achieved by RNAi, and that such a small reduction in total Aly protein (less than 25% compared to control) may not be functionally significant. Likewise, the mutant ORF57 proteins described by Nekorchuk *et* al (2007) also failed to interact with viral mRNA, which makes it difficult to interpret their significance with regards to ORF57-mediated nuclear export of viral mRNA.

### Intronless mRNA export in mammalian cells

Our findings using a naturally occurring intronless viral mRNA may provide some insight to the nuclear export of cellular intronless mRNAs, which are often studied using *in vitro*-transcribed cDNAs. The H2A intronless mRNA is exported by SRp20/9G8 that recognise and bind a specific sequence in the target intronless mRNA and subsequently promote export via a direct interaction with TAP (Fig. 7C i) [53],[54]. Our data suggest that ORF57 may function in a similar manner to cellular SR-proteins by binding to a sequence-specific region of the intronless viral mRNA. Work is currently underway in our laboratory to identify potential ORF57-target sequences in intronless viral mRNA. Conversely, it will be of interest to determine whether hTREX is recruited to H2A mRNA by SR-proteins.

More recently, Aly was shown to be recruited to an *in vitro* transcribed intronless β-globin construct independently of splicing, via a direct interaction with the CBC protein, CBP20 [55]. In contrast, a second publication suggested that Aly is recruited to *in vitro* transcribed intronless mRNA by UAP56 in ATP-dependent manner [56]. Interestingly, while there are some disparities, the observations made by both groups generally support a model whereby the 5′ cap, Aly and UAP56 are involved in intronless mammalian mRNA nuclear export (Fig. 7C ii and iii). Once again, it will be interesting to see if further analysis reveals the presence of an entire hTREX complex on these mRNAs.

In summary, these data highlight that a complete hTREX complex is required for efficient KSHV intronless mRNA export and replication. Importantly, data herein demonstrate that recruitment of the nuclear export factor TAP and its adapter protein, Aly, are not sufficient to promote nuclear export. These data suggests that UAP56 and the hTHO-complex must be recruited in order to form an export competent KSHV intronless mRNP.

## Materials and Methods

### Plasmid and Antibody details

Oligonucleotides used in cloning, RT-PCR analysis and mutagenesis can be found in Table S1. To generate pORF57GFP, ORF57 cDNA was amplified by PCR and cloned into pEGFP-N1 (BD Biosciences Ltd). pORF57GFPpmut was generated using the QuickChange II site-directed mutagenesis kit (Stratagene). pORF47 and pORFgB were cloned into pCDNA3.1+ (Invitrogen). pETORF57 and pETORF57pmut were cloned in pET21b (Novogen). The genomic ORF50 gene was cloned into pCS2MT+ [57]. To generate pGST-ORF57pmut, the NcoI/HindIII fragment of pGST-ORF57 [28] was replaced with the NcoI/HindIII fragment from pORF57GFPpmut. pAlymyc and pAlyΔmyc [42], pGST-Aly, pGST-UAP56, pGST-hHpr1 and pUAP56-His [9] and pET-CBP80 [13] have all been described elsewhere.

SRp20, Y14, and Magoh (Santa Cruz Biotech), p53 (Pharmagen Inc), GFP mAb and GFP pAb (BD Biosciences) and Myc, SC-35, B-actin and GAPDH (Sigma) were purchased from the respective suppliers. Specific antibodies to CBP80, Aly, UAP56, hHpr1, fSAP79, hTho2 and eIF4A3 were previously described [9],[13]. Unless stated all antibodies were used at a dilution of 1:1000 for western blot analysis.

### Cell culture, viruses and transfection

HEK-293T, HeLa cells and 293T BAC36 cells harbouring a recombinant KSHV BAC36 genome [41] were cultured in Dulbecco's modified Eagle medium (DMEM, Invitrogen, Paisley, UK) supplemented with 10% foetal calf serum (FCS, Invitrogen), glutamine and penicillin-streptomycin. KSHV-infected BCBL-1 cells were cultured in RPMI medium (Invitrogen, Paisley, UK) supplemented with 10% foetal calf serum (FCS, Invitrogen), glutamine and penicillin-streptomycin. 293T BAC36 cells and BCBL-1 cells were reactivated using TPA (20 ng/ml) for 24 h. Plasmid transfections were carried out using Lipofectamine™ 2000 (Invitrogen, Paisley, UK), as per the manufacturer's instructions.

### Immunoprecipitation Assays

Glutathione S-transferase (GST) pulls downs and co-immunoprecipitations, in addition to subsequent protein analysis by SDS-PAGE and western blot, were performed as previously described [29]. Confirmation of successful RNase was carried out as described by Carlile *et al* (Fig. S7) [55],[58]. A polyclonal antibody to p53 served as an unrelated antibody control throughout. This antibody precipitated the cognate p53 protein (Fig. S8). RNA-immunoprecipitations were performed as previously described [59].

### Northern Blot analysis

Nuclear and cytoplasmic RNA for northern blots was extracted from transiently transfected cells using the PARIS™ kit (Ambion Inc., Warrington, UK) as per the manufacturer's instructions. Northern blots were carried out as described previously [60]. Membrane bound RNA was hybridised with ^32^P-radiolabelled random-primed probes specific for ORF47 and 18S rRNA. The blots were then analysed using a FUJIX BAS1000 Bio-Imaging Analyser (Fuji Photo Film Co. Ltd) and data quantified using the AIDA (Advanced Image Data Analyser) version 2.31 software.

### *In situ* hybridisation

*In situ* hydridisation was performed as previously described [61]. 25 ng of biotin-labelled probes specific for KSHV ORF47 mRNA were denatured and hybridised at 37°C for 4 hrs. For detection, cells were incubated for 30 min with 7 µl of 12.5 µg/ml of Cy5-streptavidin (Molecular probes). Coverslips were mounted in Vectorshield® mounting medium (Vector Laboratories, CA) and staining visualised on an Upright LSM 510 META Axioplan 2 confocal microscope (Zeiss) using the LSM Imaging software (Zeiss).

### Virus replication assays

293-T BAC36 cells harbouring KSHV BAC36 under hygromycin selection were reactivated using 20 ng/ml TPA. At 72 h post reactivation, filtered tissue culture supernatants were used to spinoculate 1×10^5^ HEK 293-T cells in the presence of 5 µg/ml polybrene. Infected EGFP-positive cells were quantified at 48 h post-infection by fluorescence microscopy.

## Supporting Information

Table S1 List of oligonucleotides used in this study. Oligonucleotides used in this study, the noted restriction sites refer to sites included in the sequence to facilitate direct cloning of PCR products into the vectors described in Materials and Methods above.
(2.14 MB TIF)

Figure S1 KSHV ORF57 colocalises with hTREX proteins. Cells were transfected with pORF57GFP, incubated for 24 h, fixed and immunofluorescence staining performed using the indicated antibody (A–F). Bar = 5 mm. The ORF57GFP fusion protein localised to nuclear speckles and the nucleolus. A proportion of ORF57GFP was seen to co-localise with the splicing factor, SC35 and hTREX proteins; Aly, UAP56, fSAP79 and hTho2 at nuclear speckles.
(4.92 MB TIF)

Figure S2 EJC-specific antibodies immunoprecipitate other EJC components. In order to confirm that the lack of interaction observed between components of the EJC and ORF57 in Figure 1 (B–D) was not due to the EJC-specific antibodies not working in the immunoprecipitation assay, the immunoprecipitates were analysed by western blot for the presence of Magoh (in the case of the rabbit eIF4A3 pAb IP) or eIF4A3 (in the case of the mouse Y14 and Magoh IPs). As can be seen in each case the EJC-specific antibody precipitated other core members of the EJC, but not members of hTREX, confirming that the observed lack of interaction between EJC and ORF57 was not due to a failed immunoprecipitation assay.
(1.60 MB TIF)

Figure S3 Mutation of the ORF57 PxxP motif disrupts the interaction between ORF57 and Aly but does not affect other ORF57 functions. To confirm that the mutagenesis of the PxxP motif had no effect on ORF57 stability or other functional domains, several independent experiments were performed. ORF57GFP and ORF57PmutGFP were compared to determine if they differed in subcellular localisation, protein-protein interaction or RNA binding ability. (A) To determine differences in subcellular localisation, pORF57GFP and pORF57PmutGFP were transiently transfected into 293T cells and ORF57 localisation determined via direct fluorescent visualisation of GFP. (B) To determine whether both proteins could homodimerise recombinant GST and GST-ORF57 were bound to glutathione-agarose beads and incubated with 35S-Methionine-labeled ORF57 or ORF57Pmut produced by in vitro-coupled transcription/translation. Following washes, bound proteins were separated by SDS-PAGE and the gel vacuum dried. The dried gel was exposed to autoradiograph film for 16 hrs and then developed. ITT input controls for ORF57 and ORF57Pmut are shown. (C) To determine whether both proteins interacted with the KSHV ORF50 protein 293T cells were transfected with either pGFP, pORF57GFP or pORF57PmutGFP in the presence of pORF50 and immunoprecipitations performed with a KSHV ORF50-specific polyclonal antibody. Western blot analysis was carried out using a GFP-specific antibody to detect immunoprecipitated proteins. Total cell lysate from pGFP, pORF57GFP or pORF57PmutGFP-transfected 293T cells served as positive controls (input). (D) To determine whether both proteins could bind RNA 293T cells were co-transfected with pORF47 in the absence or presence of pGFP, pORF57GFP or pORF57mutGFP and incubated for 24 h. Following UV crosslinking, RNA-immunoprecipitations were performed using GFP-specific antibodies. Total RNA extracted from mock transfected and ORF47 transfected 293T cells served as controls (input).
(2.71 MB TIF)

Figure S4 ORF57GFPpmut binds directly to CBP80 and with a similar affinity to wild type ORF57. (A) Recombinant GST, GST-Aly, GST-ORF57 and GST-ORF57pmut bound to beads were separated by SDS-PAGE and proteins visualised by coomassie staining. (B) Recombinant GST, GST-Aly, GST-ORF57 and GST-ORF57pmut were bound to glutathione-agarose beads and incubated with ^35^S-Met-labeled CBP80 produced by ITT. Following washes, bound proteins were separated by SDS-PAGE and the dried gel was exposed to autoradiograph film for 16 hrs. ITT input control for CBP80 is shown.
(2.05 MB TIF)

Figure S5 Concurrent transfection/reactivation of 293T BAC36 cells with pORF57GFPpmut and TPA leads to reduced infectious virus production compared to wild type. 293T BAC36 cells were transfected with the indicated vectors and concurrently reactivated using TPA. Lytic virus replication was assayed by harvesting the supernatant of transfected 293T BAC36 cells 72 hours post transfection/reactivation. Supernatant was used to infect 293T cells and 48 h later the level of virus infection was scored by direct-immunofluorescence. Data is derived from three independent repeats, n = 3000.
(1.63 MB TIF)

Figure S6 EJC-specific antibodies immunoprecipitate other EJC components. Immunoprecipitation of endogenous EJC components fail to co-precipitate with ORF57 in HVS-infected cells. OMK cells were infected with HVS S4-A11 and after 24 h total cell lysate was extracted and used in immunoprecipitations with the labelled antibodies. Western blot analysis revealed that HVS ORF57 co-precipitates with the hTREX protein, hHpr1, but not with eIF4A3, Y14 or Magoh during lytic infection. As can be seen the Y14- and Magoh-specific antibodies co-precipitated with eIF4A3, confirming that the observed lack of interaction between these proteins and HVS ORF57 was not due to a failed immunoprecipitation assay.
(1.61 MB TIF)

Figure S7 Confirmation of efficient RNase treatment. In order to confirm that the RNase treatment of cell lystates was efficient, a RNase dependent co-immunoprecipitation was performed. It has previously been shown that the association between PML and GAPDH depends on the presence of RNA (Carlilie et al., 1998. Biochem J. 335, 691–696). 293T cell lysates were incubated for 30 mins at 37°C with RNase at a concentration of 20 µg/ml or PBS control. Immunoprecipitations were performed with an antibody specific for PML. Western blot analysis was carried out using a GAPDH-specific antibody, to detect immunoprecipitated GAPDH protein. Total cell lysate from 293T cells served as a positive control (input). These RNase conditions were used in all experiments.
(1.65 MB TIF)

Figure S8 Control p53 antibody precipitates the cognate protein but not hTREX proteins. A p53-specific antibody has been used as a negative control in all immunoprecipitation assays. To confirm this antibody was able to precipitate the cognate p53 protein immunoprecipitations were performed on 293T cell lysates using the p53-specific antibody. Western blot analysis was carried out using p53- or hTho1-specific antibodies, to detect immunoprecipitated proteins. Total cell lysate from 293T cells served as a positive control (input).
(1.74 MB TIF)
