# Supplementary figures and images for: Recruitment of the Complete hTREX Complex Is Required for Kaposi's Sarcoma–Associated Herpesvirus Intronless mRNA Nuclear Export and Virus Replication
Source: PLoS Pathog. 2008 Oct 31;4(10):e1000194. doi: 10.1371/journal.ppat.1000194 (PMC2569588; doi:10.1371/journal.ppat.1000194)

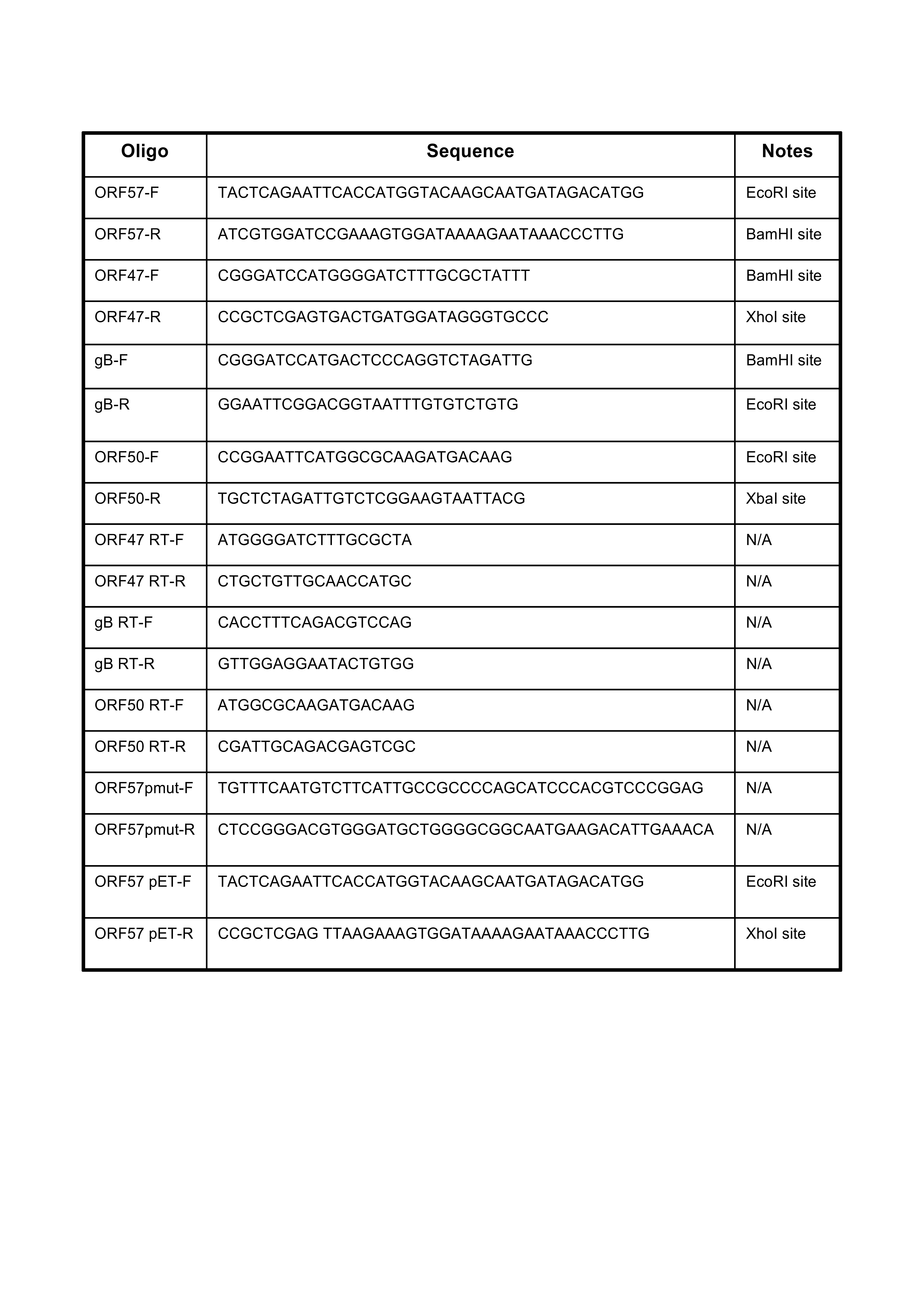

Supplement: Table S1 — List of oligonucleotides used in this study. Oligonucleotides used in this study, the noted restriction sites refer to sites included in the sequence to facilitate direct cloning of PCR products into the vectors described in Materials and Methods above. (2.14 MB TIF) [file ppat.1000194.s001.tif]

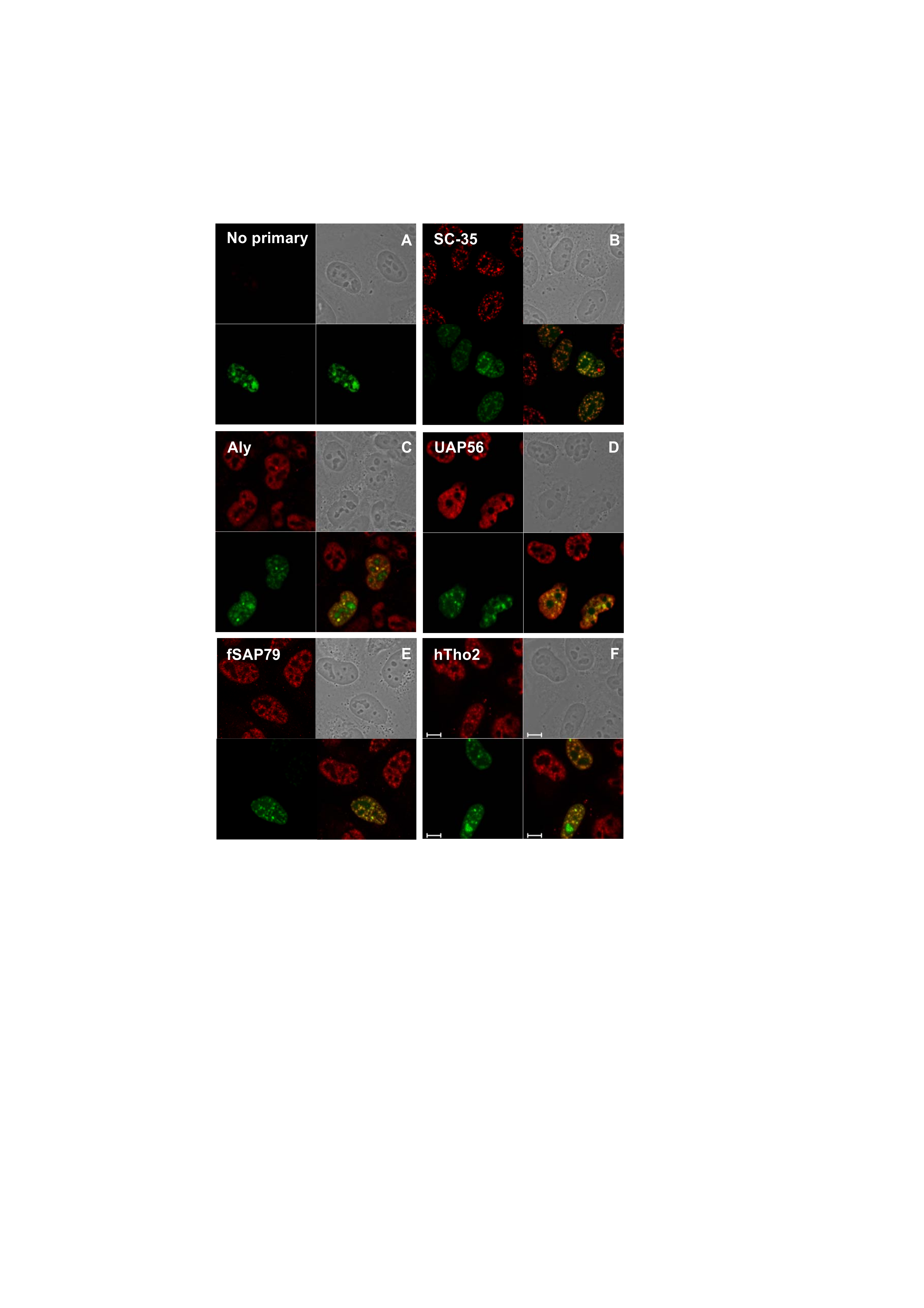

Supplement: Figure S1 — KSHV ORF57 colocalises with hTREX proteins. Cells were transfected with pORF57GFP, incubated for 24 h, fixed and immunofluorescence staining performed using the indicated antibody (A–F). Bar = 5 mm. The ORF57GFP fusion protein localised to nuclear speckles and the nucleolus. A proportion of ORF57GFP was seen to co-localise with the splicing factor, SC35 and hTREX proteins; Aly, UAP56, fSAP79 and hTho2 at nuclear speckles. (4.92 MB TIF) [file ppat.1000194.s002.tif]

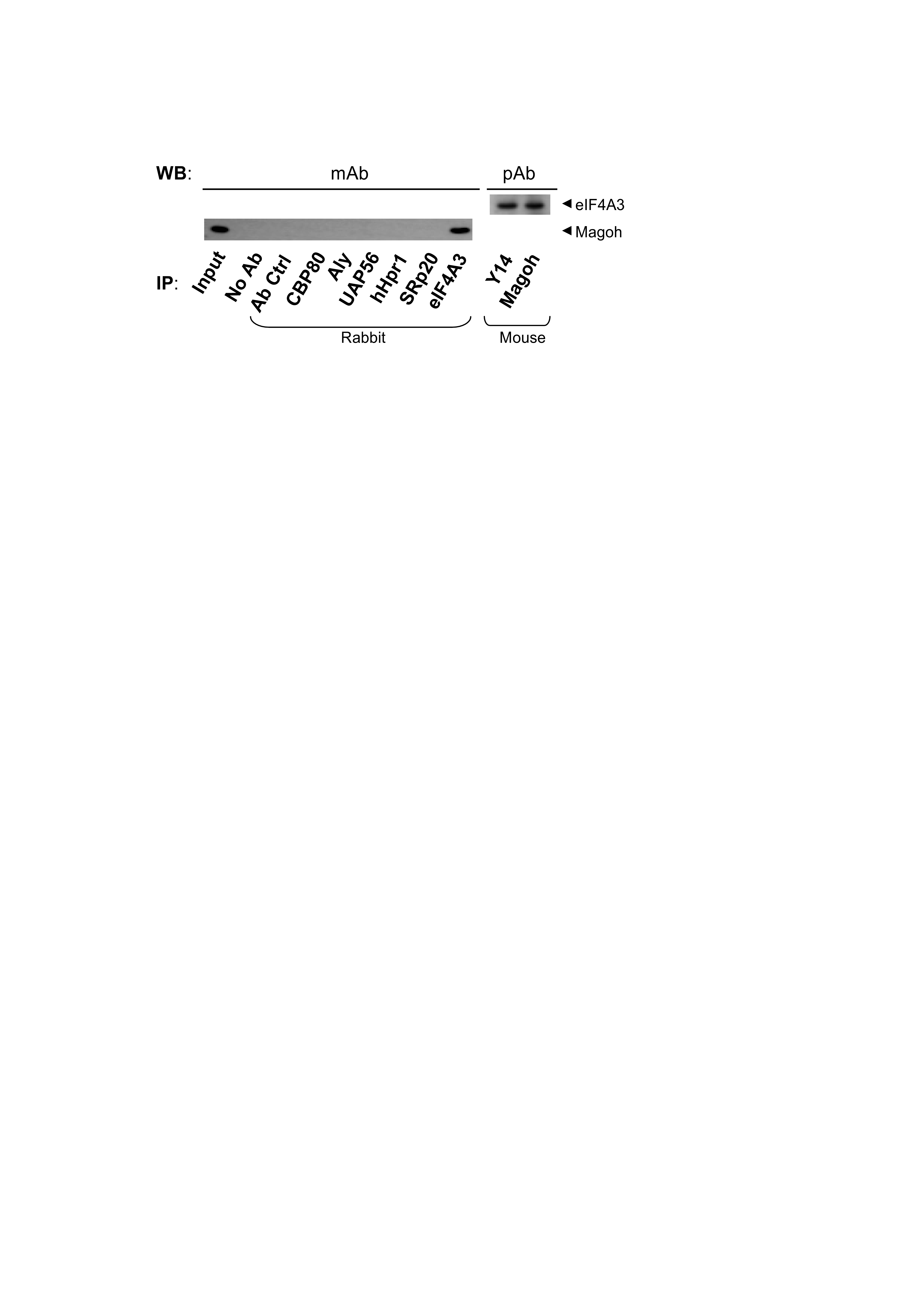

Supplement: Figure S2 — EJC-specific antibodies immunoprecipitate other EJC components. In order to confirm that the lack of interaction observed between components of the EJC and ORF57 in Figure 1 (B–D) was not due to the EJC-specific antibodies not working in the immunoprecipitation assay, the immunoprecipitates were analysed by western blot for the presence of Magoh (in the case of the rabbit eIF4A3 pAb IP) or eIF4A3 (in the case of the mouse Y14 and Magoh IPs). As can be seen in each case the EJC-specific antibody precipitated other core members of the EJC, but not members of hTREX, confirming that the observed lack of interaction between EJC and ORF57 was not due to a failed immunoprecipitation assay. (1.60 MB TIF) [file ppat.1000194.s003.tif]

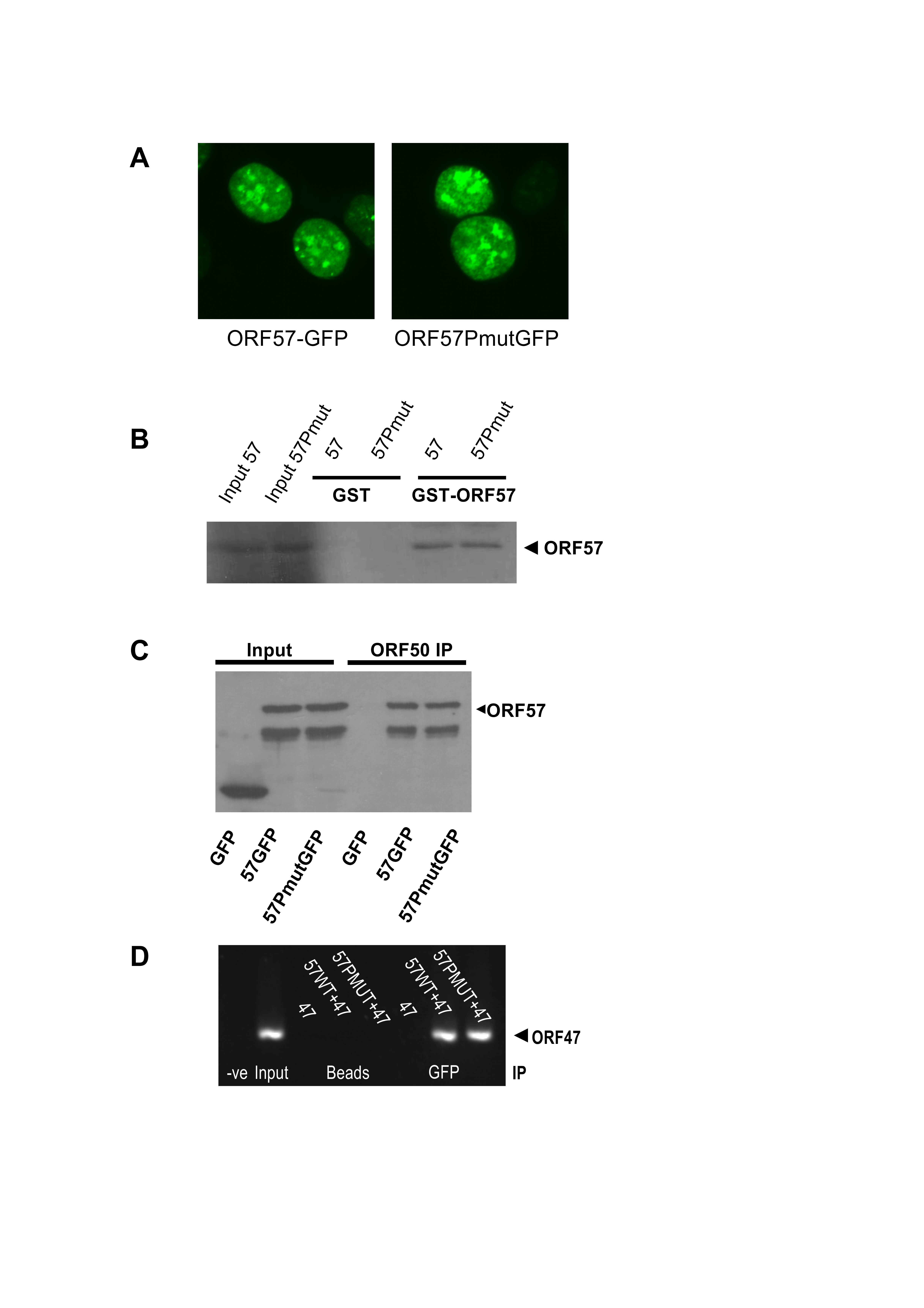

Supplement: Figure S3 — Mutation of the ORF57 PxxP motif disrupts the interaction between ORF57 and Aly but does not affect other ORF57 functions. To confirm that the mutagenesis of the PxxP motif had no effect on ORF57 stability or other functional domains, several independent experiments were performed. ORF57GFP and ORF57PmutGFP were compared to determine if they differed in subcellular localisation, protein-protein interaction or RNA binding ability. (A) To determine differences in subcellular localisation, pORF57GFP and pORF57PmutGFP were transiently transfected into 293T cells and ORF57 localisation determined via direct fluorescent visualisation of GFP. (B) To determine whether both proteins could homodimerise recombinant GST and GST-ORF57 were bound to glutathione-agarose beads and incubated with 35S-Methionine-labeled ORF57 or ORF57Pmut produced by in vitro-coupled transcription/translation. Following washes, bound proteins were separated by SDS-PAGE and the gel vacuum dried. The dried gel was exposed to autoradiograph film for 16 hrs and then developed. ITT input controls for ORF57 and ORF57Pmut are shown. (C) To determine whether both proteins interacted with the KSHV ORF50 protein 293T cells were transfected with either pGFP, pORF57GFP or pORF57PmutGFP in the presence of pORF50 and immunoprecipitations performed with a KSHV ORF50-specific polyclonal antibody. Western blot analysis was carried out using a GFP-specific antibody to detect immunoprecipitated proteins. Total cell lysate from pGFP, pORF57GFP or pORF57PmutGFP-transfected 293T cells served as positive controls (input). (D) To determine whether both proteins could bind RNA 293T cells were co-transfected with pORF47 in the absence or presence of pGFP, pORF57GFP or pORF57mutGFP and incubated for 24 h. Following UV crosslinking, RNA-immunoprecipitations were performed using GFP-specific antibodies. Total RNA extracted from mock transfected and ORF47 transfected 293T cells served as controls (input). (2.71 MB TIF) [file ppat.1000194.s004.tif]

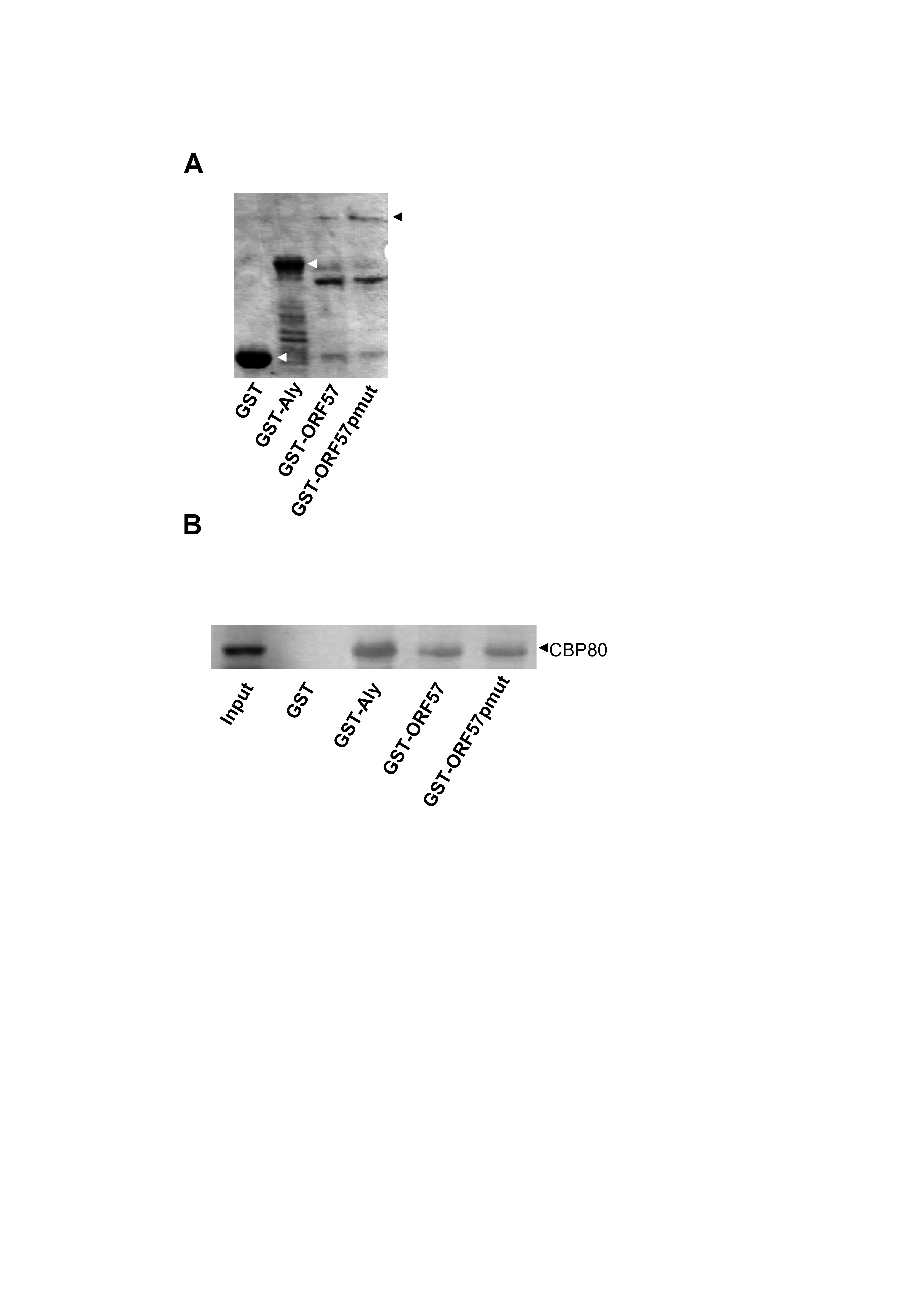

Supplement: Figure S4 — ORF57GFPpmut binds directly to CBP80 and with a similar affinity to wild type ORF57. (A) Recombinant GST, GST-Aly, GST-ORF57 and GST-ORF57pmut bound to beads were separated by SDS-PAGE and proteins visualised by coomassie staining. (B) Recombinant GST, GST-Aly, GST-ORF57 and GST-ORF57pmut were bound to glutathione-agarose beads and incubated with 35S-Met-labeled CBP80 produced by ITT. Following washes, bound proteins were separated by SDS-PAGE and the dried gel was exposed to autoradiograph film for 16 hrs. ITT input control for CBP80 is shown. (2.05 MB TIF) [file ppat.1000194.s005.tif]

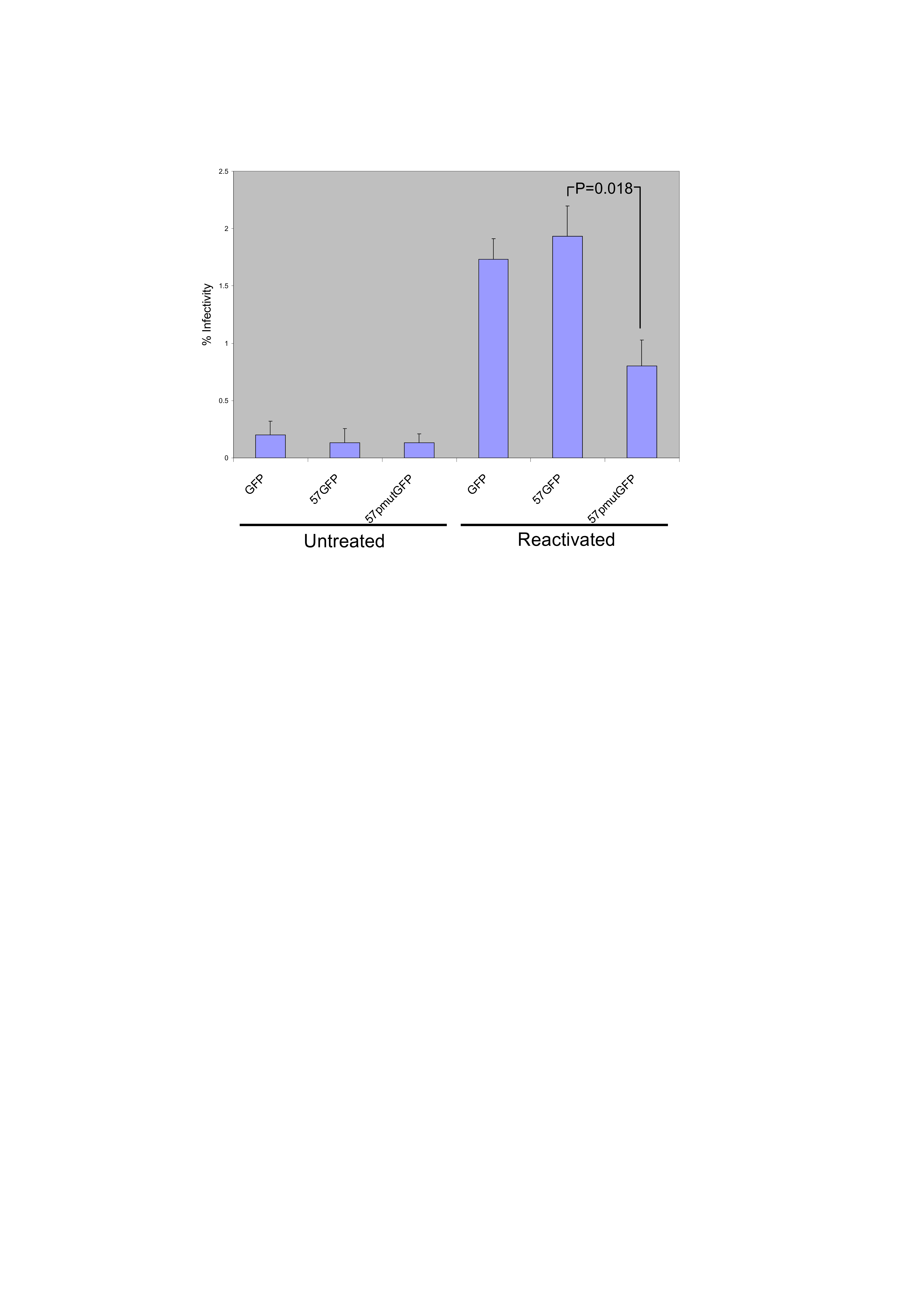

Supplement: Figure S5 — Concurrent transfection/reactivation of 293T BAC36 cells with pORF57GFPpmut and TPA leads to reduced infectious virus production compared to wild type. 293T BAC36 cells were transfected with the indicated vectors and concurrently reactivated using TPA. Lytic virus replication was assayed by harvesting the supernatant of transfected 293T BAC36 cells 72 hours post transfection/reactivation. Supernatant was used to infect 293T cells and 48 h later the level of virus infection was scored by direct-immunofluorescence. Data is derived from three independent repeats, n = 3000. (1.63 MB TIF) [file ppat.1000194.s006.tif]

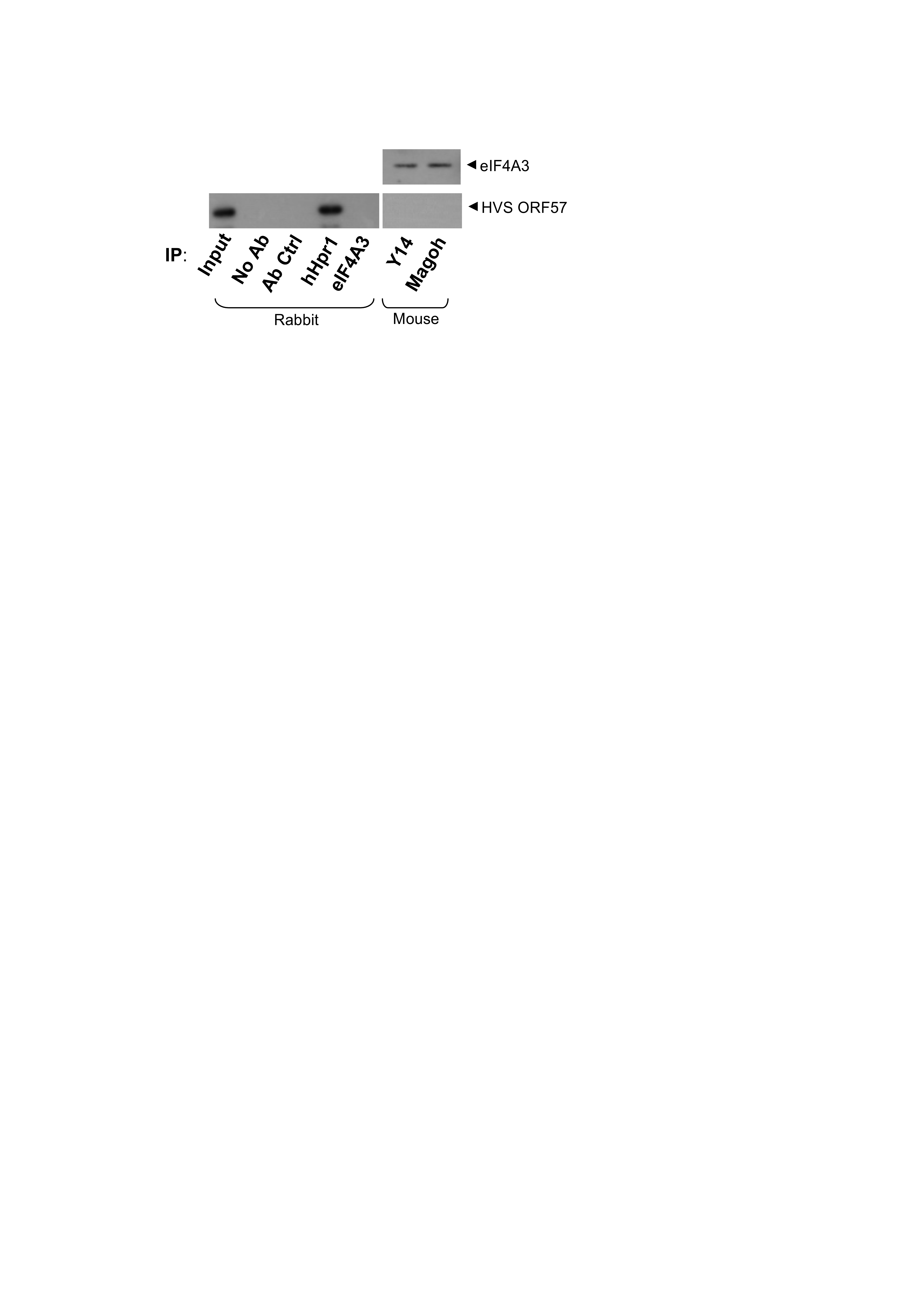

Supplement: Figure S6 — EJC-specific antibodies immunoprecipitate other EJC components. Immunoprecipitation of endogenous EJC components fail to co-precipitate with ORF57 in HVS-infected cells. OMK cells were infected with HVS S4-A11 and after 24 h total cell lysate was extracted and used in immunoprecipitations with the labelled antibodies. Western blot analysis revealed that HVS ORF57 co-precipitates with the hTREX protein, hHpr1, but not with eIF4A3, Y14 or Magoh during lytic infection. As can be seen the Y14- and Magoh-specific antibodies co-precipitated with eIF4A3, confirming that the observed lack of interaction between these proteins and HVS ORF57 was not due to a failed immunoprecipitation assay. (1.61 MB TIF) [file ppat.1000194.s007.tif]

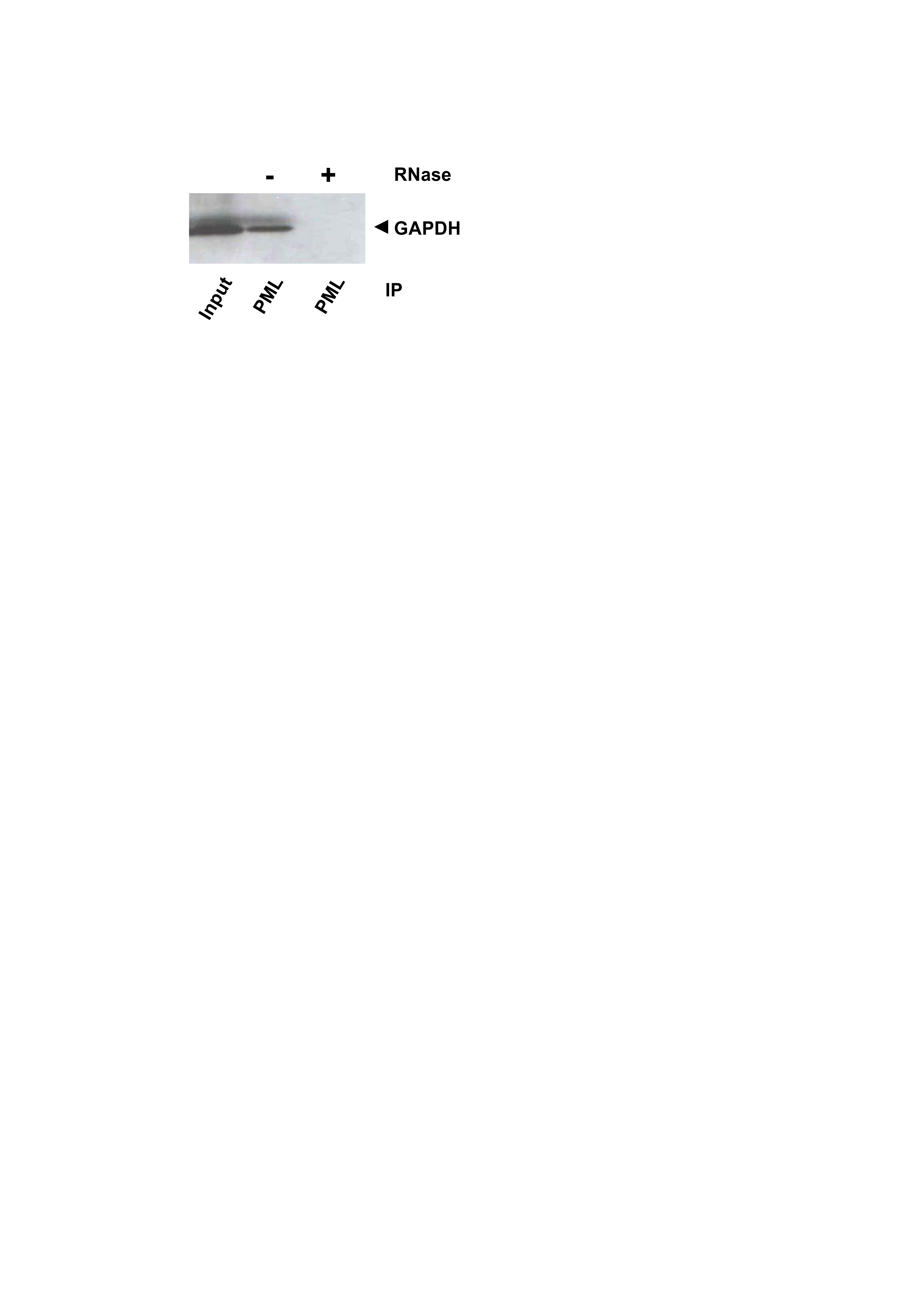

Supplement: Figure S7 — Confirmation of efficient RNase treatment. In order to confirm that the RNase treatment of cell lystates was efficient, a RNase dependent co-immunoprecipitation was performed. It has previously been shown that the association between PML and GAPDH depends on the presence of RNA (Carlilie et al., 1998. Biochem J. 335, 691–696). 293T cell lysates were incubated for 30 mins at 37°C with RNase at a concentration of 20 µg/ml or PBS control. Immunoprecipitations were performed with an antibody specific for PML. Western blot analysis was carried out using a GAPDH-specific antibody, to detect immunoprecipitated GAPDH protein. Total cell lysate from 293T cells served as a positive control (input). These RNase conditions were used in all experiments. (1.65 MB TIF) [file ppat.1000194.s008.tif]

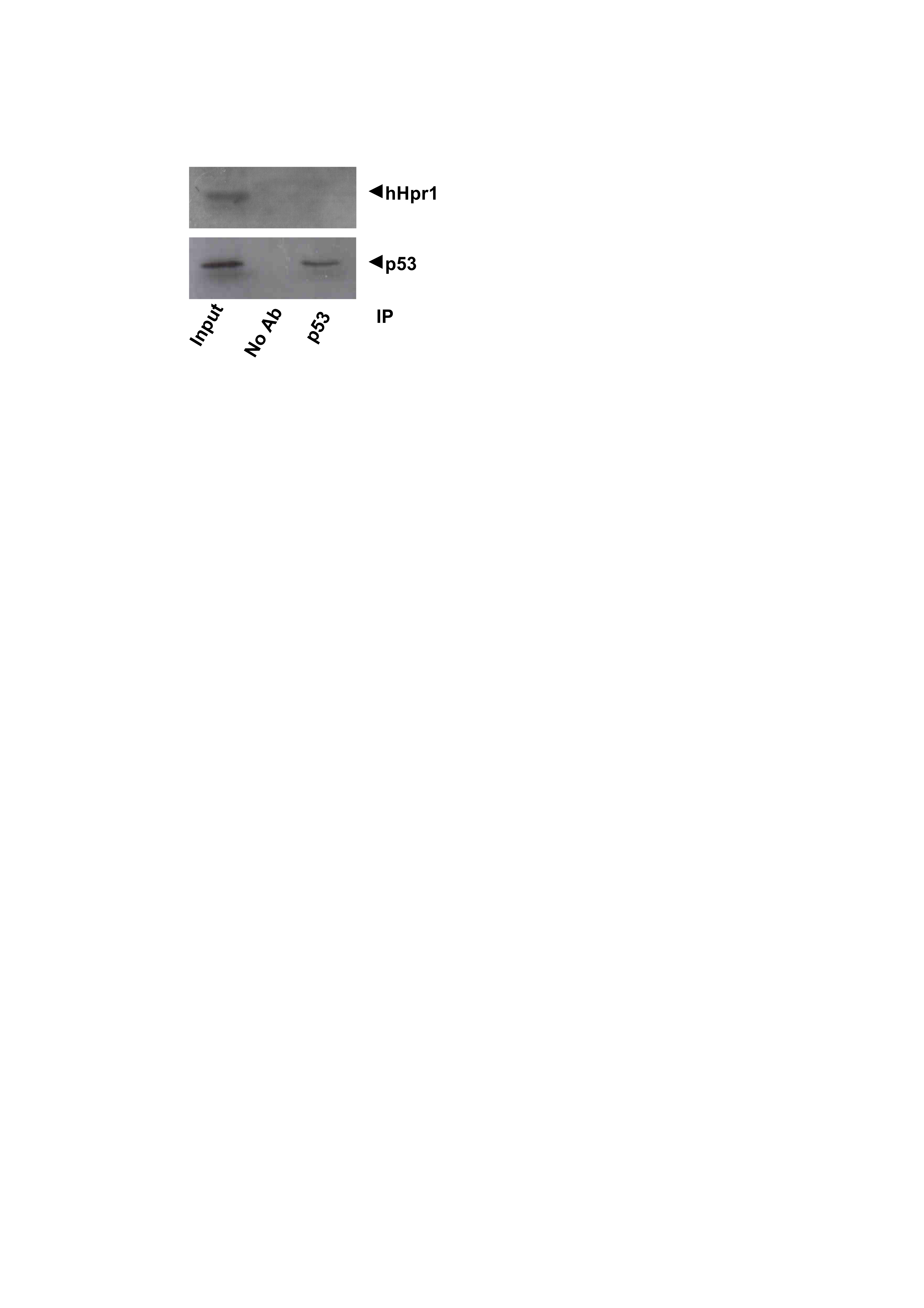

Supplement: Figure S8 — Control p53 antibody precipitates the cognate protein but not hTREX proteins. A p53-specific antibody has been used as a negative control in all immunoprecipitation assays. To confirm this antibody was able to precipitate the cognate p53 protein immunoprecipitations were performed on 293T cell lysates using the p53-specific antibody. Western blot analysis was carried out using p53- or hTho1-specific antibodies, to detect immunoprecipitated proteins. Total cell lysate from 293T cells served as a positive control (input). (1.74 MB TIF) [file ppat.1000194.s009.tif]
